# Deriving Mental Energy From Task Completion

**DOI:** 10.3389/fpsyg.2021.717414

**Published:** 2021-08-20

**Authors:** Xiang Wang, Chris Janiszewski, Yanmei Zheng, Juliano Laran, Wonseok Eric Jang

**Affiliations:** ^1^Warrington College of Business, University of Florida, Gainesville, FL, United States; ^2^Shidler College of Business, University of Hawaii at Manoa, Honolulu, HI, United States; ^3^Faculty of Business and Economics, University of Basel, Basel, Switzerland; ^4^College of Sports Science, Sungkyunkwan University, Suwon, South Korea

**Keywords:** cognitive resources, mental energy, task rewards, task completion, extrinsic motivation

## Abstract

Many tasks in everyday life (e.g., making an accurate decision, completing job tasks, and searching for product information) are extrinsically motivated (i.e., the task is performed to gain a benefit) and require mental effort. Prior research shows that the cognitive resources needed to perform an extrinsically motivated task are allocated pre-task. The pre-task allocation of mental resources tends to be conservative, because mental effort is costly. Consequently, there are mental energy deficits when the use of mental resources exceeds the allocated amount. This research provides evidence for post-task mental energy replenishment. The amount of resource replenishment is a function of the size of the mental energy deficit and the favorability of the cost-benefit trade-off experienced at the completion of the task (i.e., the value of the reward given the energy investment). The findings have implications for how cognitive resources management influences the availability of mental energy on a moment-to-moment basis.

## Introduction

Cognitive resources are a foundational concept in the cognitive sciences. Cognitive resource availability influences perception, comprehension, and elaboration in information processing models (Greenwald and Leavitt, 1984; Wingfield, 2016), the ability to engage in system 2 processes (e.g., rule-based reasoning, analytic thought, and planning) in dual-process models (Evans, 2008), and the effectiveness of behavior in models of self-control (Inzlicht et al., 2021). Exerting more cognitive effort improves decision accuracy (Bettman et al., 1998) and the effectiveness of behavior (Shenhav et al., 2017).

**Figure 1:**
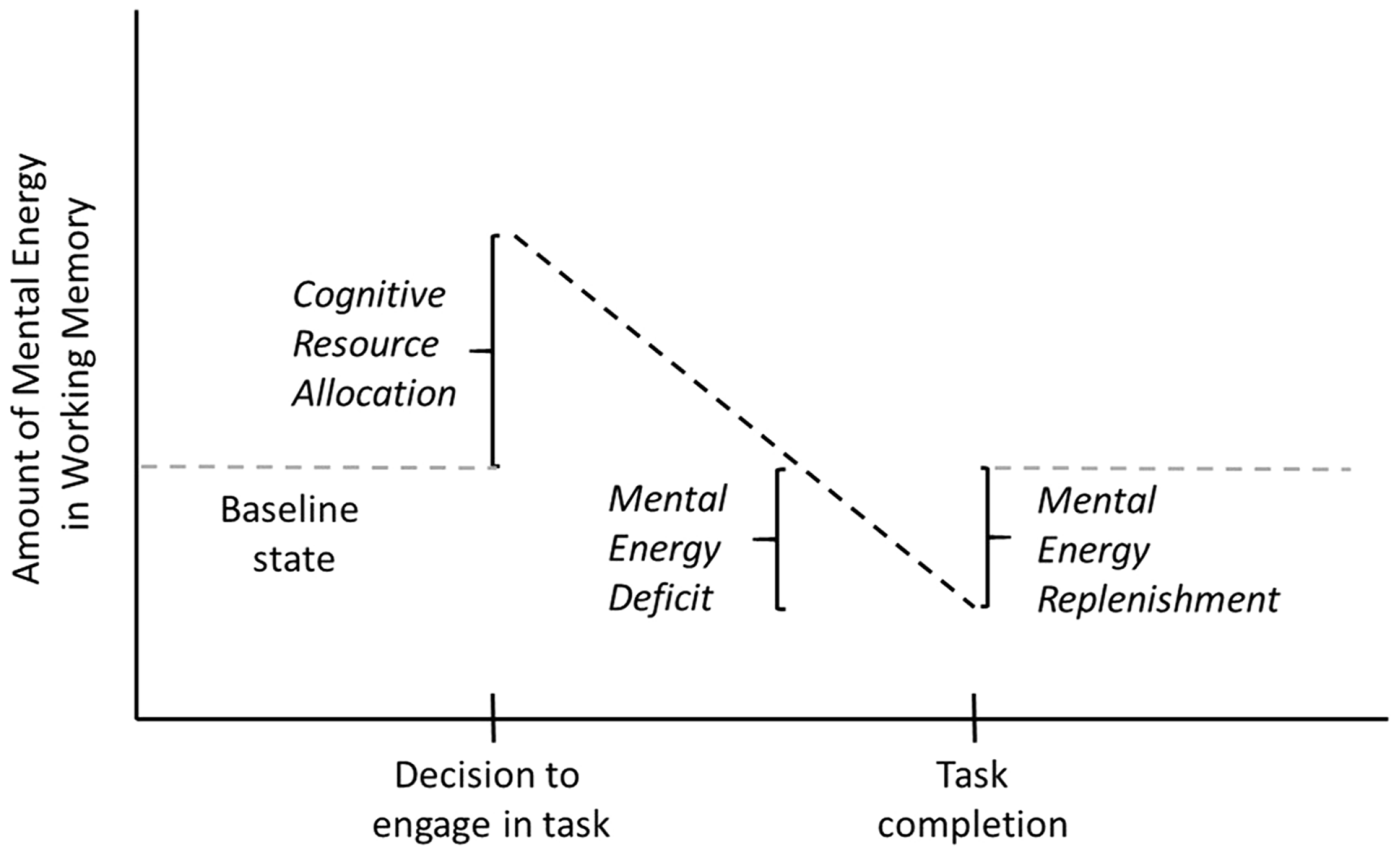
Illustration of cognitive resource allocation, a mental energy deficit, and replenishment. The baseline level of mental energy is shown by the gray dashed line. The decision to engage in a task increases the level of mental energy (cognitive resource allocation). The amount of mental energy used during the completion of a task can be more than was allocated (mental energy deficit). Task completion provides an opportunity for mental energy replenishment.

Resource-based models of cognition and behavior assume people expend cognitive resources in order to achieve a beneficial outcome, whether that outcome be a better decision, a more effective behavior, or a more rewarding consumption experience (Shenhav et al., 2017). Prior to engaging in a task, people assess the amount of cognitive effort (i.e., costs) needed to complete the task and the benefits that can be accrued from task completion (Brehm and Self, 1989; Boksem and Tops, 2008; Shenhav et al., 2017; Kool and Botvinick, 2018). If the anticipated benefits exceed the anticipated costs, people allocate cognitive resources into working memory (henceforth, mental energy) and engage in the task (see Figure 1; Navon and Gopher, 1979; Brehm and Self, 1989; Botvinick and Braver, 2015; Shenhav et al., 2017). During task engagement, mental energy is expended to enhance mental focus (i.e., task performance) and facilitate mental intensity (i.e., task persistence; Shenhav et al., 2013).

In this paper, we ask the question, “What if a task is more demanding than expected?” If the actual cognitive resources needed to perform a task exceed the cognitive resources allocated for the task, post-task mental energy will be in a deficit state relative to baseline (i.e., the mental energy level prior to considering the task; see Figure 1). If mental energy allocation only occurs pre-task, and only depends on task characteristics, then sequences of tasks that result in an energy deficit should lead to degradations in cognitive performance. Accordingly, we posit that an adaptive response has emerged wherein automatic, post-task mental energy allocation can address a mental energy deficit. We further posit that this adaptive response should be sensitive to two factors: (1) the size of the mental energy deficit and (2) the favorability of the cost-benefit trade-off experienced at the completion of the task. Specifically, unexpected cognitive effort creates a mental energy deficit and a need to replenish the energy (Jansen et al., 2002, 2003; van Veldhoven and Broersen, 2003). A favorable cost-benefit trade-off (i.e., the task benefits are sufficient given the actual mental energy costs) will result in mental energy replenishment, whereas an unfavorable trade-off (i.e., the task benefits are insufficient given the actual mental energy costs) will not result in mental energy replenishment. In the latter case, the mental energy deficit acts as a signal that effort is being poorly invested and that corrective action should be taken (e.g., disengage from task, update priors about the mental energy requirements for the type of task performed, rest).

This research provides a more nuanced explanation of how mental energy is managed on a task-to-task basis and provides two insights into mental energy supplies. First, we know that mental energy varies on a moment-to-moment basis (Yeo and Neal, 2008). Yet, prior conceptualizations of cognitive resource management do not address the drivers of moment-to-moment changes in mental energy availability (Yeo and Neal, 2008; Shenhav et al., 2017). Our work provides insight into how unexpected effort affects (i.e., decreases and increases) mental energy, which in turn can influence performance on subsequent cognitive tasks. Second, the results provide insight into why people are cognitive misers (Navon and Gopher, 1979; Goldfarb and Henik, 2014; Kool and Botvinick, 2014; Sayalı and Badre, 2019). In general, people allocate the minimum amount of cognitive resources needed to complete a task. Consequently, resource allocation errors are primarily negative (i.e., there is a tendency toward an under allocation of resources). A resource allocation system characterized by under allocation can only be sustainable if there is a post-task correction mechanism. In the absence of this mechanism, the predominant experience of most people would be a perpetual deficit in mental energy. Our work provides insight into why this is not the case.

## Theory and Hypotheses

### Mental Energy

Mental energy has been conceptualized “as a subjective feeling about one’s capacity to accomplish tasks in daily life” (O’Connor, 2006a). Within this conceptualization, mental energy is multi-dimensional construct consisting of (1) the mood of energy (i.e., the feeling that one can complete physical and mental tasks), (2) motivation (i.e., the desire to execute tasks), (3) cognitive resources (i.e., the ability to execute of cognitive tasks), and (4) quality of life (i.e., the degree to which life tasks are accomplished; O’Connor, 2006a). Mental energy researchers have focused primarily on the mood of energy and cognitive resource dimensions, with motivation being studied primarily in the goal literature and quality of life being studied primarily in the social welfare literature.

A considerable amount of research has focused on mental energy as a mood or feeling (O’Connor, 2004, 2006b; Lieberman, 2006). The *feeling of mental energy* is a general feeling that one is able to complete mental or physical activities (O’Connor, 2006b; Boolani et al., 2020). Common measures of the feeling of mental energy include the single-item visual analog scale (Wood and Magnello, 1992), the profile of mood states short form (“energetic,” “full of pep,” “vigorous,” “active,” and “lively;” Heuchert and McNair, 2012), and the mental energy state and trait scale (O’Connor, 2006b). The feeling of mental energy is impacted by sleep duration (Boolani and Manierre, 2019), time of day (Wood and Magnello, 1992), resistance exercise (Ward-Ritacco et al., 2016), illness (Loy et al., 2018), and food consumption (Lieberman, 2007; Maridakis et al., 2009), among other things. Experimental evidence shows that increasing this feeling increases vigilance (Maridakis et al., 2009) and decreases balance in the elderly (Boolani et al., 2020).

Considerable attention has also been devoted to investigating mental energy as a cognitive resource (James, 1907; Carver and Scheier, 1981; Lieberman, 2007; Goldfarb and Henik, 2014; Shenhav et al., 2017). The *mental energy as a cognitive resource* literature considers the amount of mental energy available to perform a cognitive task (e.g., mental energy at the moment). Mental energy enables an executive control mechanism that “regulate(s) perceptual and motor processes in order to respond … to novel or changing task demands” (van der Linden et al., 2003, p. 47). This is especially true when people have to sustain engagement in a complex task that requires sustained attention, challenging analyses, dynamic planning, or disambiguating information (Broadbent, 1979; Bodenhausen and Lichtenstein, 1987; Campbell, 1988; Hockey, 1993; O’Connor, 2006a). In this perspective, changes in the availability of mental energy are inferred from changes in-task performance (Lieberman, 2007), including sustained attention (Schmeichel et al., 2003), organizing and evaluating information (Vohs et al., 2014), resolving choice trade-offs (Wang et al., 2010), compliance behaviors (Laran and Janiszewski, 2011), emotion regulation (Schmeichel et al., 2003), and impression management (Vohs et al., 2005). We investigate the mental energy as a cognitive resource dimension in this work.

### Pre-task Allocation of Mental Energy Resources

The extant literature proposes that cognitive resources (i.e., mental energy) are allocated to working memory prior to engaging in an extrinsically motivated task (Brehm and Self, 1989; Boksem and Tops, 2008; Shenhav et al., 2017; Kool and Botvinick, 2018). There are three factors that influence the pre-task allocation of cognitive resources to working memory: the difficulty of the task (costs), the size of the benefit (rewards), and the cost-benefit trade-off.

#### Task Difficulty

A number of theories posit that expected task difficulty influences the amount of mental energy made available prior to initiating task pursuit. The theory of motivational intensity proposes that the amount of mental energy available prior to a task will increase as the expected difficulty of a task increases, but that mental energy will decline as it becomes apparent that a task is impossible to perform (Brehm et al., 1983; Brehm and Self, 1989). Similarly, goal-setting theory assumes that more aggressive goals require more mental energy for goal pursuit (Latham et al., 2011). Supporting this idea, working memory functions better when people can anticipate the difficulty of a task, suggesting that a difficulty cue allows a person to prepare for the task by allocating more cognitive resources to working memory (Manelis and Reder, 2015).

#### Task Benefits

A number of theories propose that the expected benefits associated with completing a task influence the amount of mental energy made available prior to engaging in task pursuit. For example, drive-reduction theory assumes that the motivation (i.e., the allocation of mental and physical energy) to pursue a task is a direct function of the reward potential of a behavior (Hull, 1943). Incentive theories of motivation propose that people will work harder for more positive outcomes (Bolles, 1972; Bindra, 1974; Wigfield and Eccles, 2000). Goal-systems theory assumes that motivated goal pursuit depends on the appeal and importance of the goal outcome (Kruglanski et al., 2002). The biopsychological theory of personality assumes that a behavioral activation system energizes behavior in accordance with a person’s ability to appreciate the reward value of a behavior (Gray, 1970; Carver and White, 1994). In each model, more appealing, rewarding, or important goal outcomes generate more desire to engaging in the task, the implication being that more mental energy is available.

#### Cost-Benefit Analysis of the Task

A third factor that influences cognitive resource allocation is an analysis of the mental costs of engaging in a task vs. the benefits of task completion. Cognitive resources are allocated only when the reward is sufficient. This conceptualization assumes that mental effort is costly (Kahneman, 1973; Kurzban, 2016; Shenhav et al., 2017; Kool and Botvinick, 2018); hence, people are motivated to conserve cognitive resources (Navon and Gopher, 1979; McGuire and Botvinick, 2010; Kool and Botvinick, 2014; Dunn et al., 2016; Sayalı and Badre, 2019). People allocate the minimum amount of resources needed to complete a task, not the maximum amount of resources given the potential reward (Goldfarb and Henik, 2014). For example, cognitive energetics theory (Kruglanski et al., 2012) proposes that an allocation of cognitive resources should be equal to the “restraining force” – the resistance determined by task demands, the pull of competing goals, and one’s inclination to conserve resources (i.e., allocate the minimal amount of resources to get the task done), provided that the magnitude of “restraining force” is lower than that of the “potential driving force,” a function of goal importance and one’s cognitive capacity. Similarly, the expected value of control (EVC) theory (Shenhav et al., 2013) proposes that the dorsal anterior cingulate cortex integrates information about the expected rewards and costs of a task to estimate its EVC and determine “whether it is worth investing control [effort] in a task, how much should be invested and, when several potential tasks are in contention, which is the most worthwhile.”

Importantly, cost-benefit analyses that modulate the choice of tasks and the allocation of cognitive resources are considered to be subconscious (Boksem and Tops, 2008; Kurzban et al., 2013; Evans et al., 2016). Moreover, a growing literature suggests that a cost-benefit approach to choosing action is an adaptive advantage because it motivates behavior toward more rewarding activities and away from less rewarding ones (Boksem and Tops, 2008; Kool et al., 2010; Kurzban et al., 2013; Shenhav et al., 2013).

### Post-task Mental Energy Replenishment

Existing accounts of the pre-task allocation of cognitive resources to working memory are not able to address how people avoid the cumulative effects of mental energy deficits. If the pre-task resource allocation system is designed to conserve cognitive resources (because mental effort is costly), then there will be more under allocation than over allocation of resources in a given time period. The under allocation of cognitive resources will inevitably lead to a deficit of mental energy and diminished cognitive performance. Thus, it would be advantageous for people to have a post-task mental energy replenishment system. A post-task mental energy replenishment system may not eliminate a mental energy deficit, but it would mitigate it.

Mental energy deficits are a common outcome in a conservative mental energy allocation system. The challenge for such a system is to determine how to address each mental energy deficit. We propose that post-task mental energy replenishment is one solution. Post-task mental energy replenishment is more likely to occur when the actual effort-reward trade-off is favorable. That is, if an accurate allocation of resources would still have resulted in engaging in the task (the reward justified the actual amount of mental energy invested), then the mental energy deficit will be replenished. Replenishment occurs because the error in pre-task mental energy allocation is acceptable given the reward. In contrast, when the actual effort-reward trade-off is unfavorable, mental energy replenishment should not occur. If an accurate estimate of the cognitive resources needed for the task would have resulted in rejecting the task or engaging in other tasks (the reward does not justify the unexpected amount of energy used), then the mental energy deficit should be a signal that cognitive resources estimates were miscalibrated and corrective action should be taken (e.g., update priors about energy requirements for this type of task, rest, and reassess behavior).

To illustrate these ideas, consider a situation where a person is shopping online. The person finds an acceptable product at a major retailer. She then determines it is worthwhile to invest additional cognitive effort in searching for a better deal (i.e., the anticipated benefits of additional search exceed the anticipated cognitive costs), allocates an appropriate level of cognitive resources, and engages in the search. If the search is more difficult than expected, there will be a mental energy deficit at the conclusion of the search. Post-task mental energy replenishment will occur if the reward (realized savings over original price) is sufficient given the actual amount of effort (i.e., the actual cost-benefit trade-off is favorable). Mental energy replenishment will not occur if the reward is insufficient given the actual amount of effort (i.e., the actual cost-benefit trade-off is unfavorable). An insufficient cost-benefit trade-off can occur because the unexpected amount of effort was too extensive (i.e., the additional search required much more cognitive effort than expected) or the reward was too small (i.e., the savings were minor).

**Figure 2:**
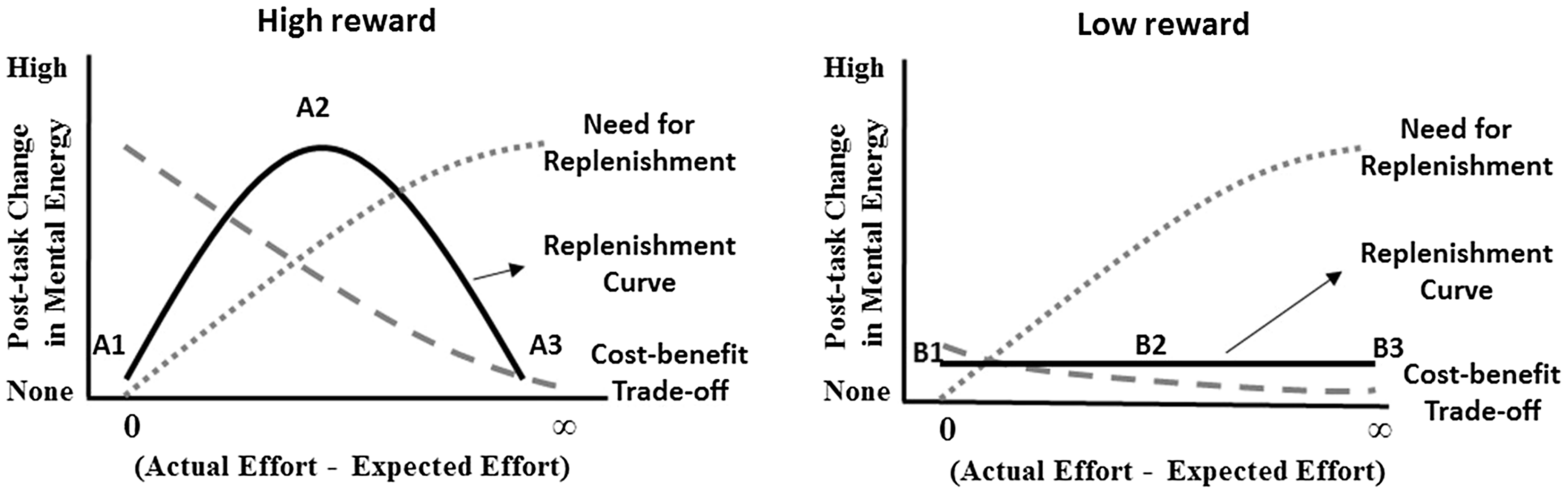
Mental energy replenishment for extrinsically motivated tasks.

As illustrated in the example, there are two forces that drive mental energy replenishment: the need for mental energy replenishment and the favorability of the cost-benefit trade-off (see Figure 2, high reward). First, unexpected effort creates a mental energy deficit and a need to replenish mental energy (Jansen et al., 2002, 2003; van Veldhoven and Broersen, 2003). The larger the amount of unexpected effort, the greater the need to replenish (Jansen et al., 2002, 2003). Second, replenishment should be strategic – it should be sensitive to the favorability of the actual cost-benefit trade-off from the completed task. This claim is consistent with the finding that people reinvest in tasks that are, on balance, rewarding (Boksem and Tops, 2008; Kool et al., 2010; Kurzban et al., 2013; Shenhav et al., 2013). An integration of the need for replenishment and favorability of the cost-benefit trade-off vectors predicts that energy replenishment will be an inverted-U function of the amount of unexpected effort when rewards are high (see the solid curve in Figure 2, high reward). Replenishment will not occur when unexpected effort is too low because the need to replenish would be negligible (see A1 in Figure 2, high reward) or when unexpected effort is too high because the cost-benefit trade-off would be unfavorable (see A3 in Figure 2, high reward). Replenishment occurs when unexpected effort is moderate because there is some need for replenishment and the cost-benefit trade-off would be favorable given the high rewards (see A2 in Figure 2, high reward). When rewards are low, the favorability of the cost-benefit trade-off declines because the rewards are less likely to be seen as worth the extra investment of effort, and thus, energy replenishment will be low (see the solid curve B1-B2-B3 in Figure 2, low reward).

Given that we are the first to propose post-task mental energy replenishment, there is little literature directly supporting the idea. Instead, one must assess if the predictions are consistent with how a conservative, pre-task cognitive resource allocation system would operate. The system we propose can not only guard against an insufficient amount of mental energy, but it can also help correct large energy allocation errors. Large mental energy deficits create a strong motivation to replenish. Consequently, a simple mental energy replenishment system could replenish energy any time there was a high need. Yet, this approach would not allow the system to learn – there would be no feedback. A better system, the one we propose, inhibits automatic mental energy replenishment when there are large energy investment errors. Unexpectedly large mental energy deficits are a signal that priors about the expected costs of completing a task need to be updated, as the anticipated effort for this type of task is highly miscalibrated (Inzlicht et al., 2015). Further, large deficits may signal that the present behavior should be abandoned or changed (Boksem and Tops, 2008; Kurzban et al., 2013). A mental energy deficit could even signal the need to switch from performing externally rewarding tasks to engaging in more intrinsically motivated activities (Inzlicht et al., 2015).

## Studies

We conducted four studies to test our predictions. Studies 1, 3, and 4 directly measured mental energy replenishment and study 2 assessed mental energy replenishment *via* performance on a subsequent task. Study 1 showed mental energy replenishment after completing a high-reward task (i.e., A2 in Figure 2), but not after completing a low-reward task (i.e., B2 in Figure 2). Study 2 used a design similar to study 1 to show that mental energy replenishment can influence performance on a subsequent task. Study 3 manipulated expected effort and reward to show that a high reward increases mental energy replenishment when the amount of unexpected effort is moderate (i.e., A2 in Figure 2), but not when it is low (i.e., A1 in Figure 2). Study 4 manipulated actual effort and reward to show an inverted-U pattern of mental energy replenishment across different levels of effort when the reward is high (i.e., A1 vs. A2 vs. A3 in Figure 2), but not when the reward is low (i.e., B1 vs. B2 vs. B3 in Figure 2).

### Study 1

The purpose of study 1 was to demonstrate that, when there is unexpected effort, mental energy is replenished upon completion of a high-reward task, but not a low-reward task. The procedure simulated online shopping behavior. The task involved finding online deals for five products, where reward value was manipulated by varying the bonus associated with finding deals. Participants were asked to find and record the deals. We predicted that participants in the high-reward condition would show mental energy replenishment at the completion of the shopping trip (i.e., A2 in Figure 2), but that participants in the low-reward condition would not (i.e., B2 in Figure 2).

#### Method

##### Participants and Design

The experiment used a two cell (reward value: low vs. high) between-subject design. An *a priori* power analysis using G*Power 3.1 (Faul et al., 2009) determined that at least 90 participants would be required to detect a medium-to-large effect (*f* = 0.30) with a power of 80%. We targeted a total sample of 100 on Amazon Mechanical Turk, and 101 participants completed the study in exchange for $1.20 in financial compensation (*M*_age_ = 32.99, 58.4% male). All participants completed the task correctly, likely because they were “master workers.” Thus, no participants were removed from the analysis.

##### Procedure and Stimuli

The study took place in mid-December, during the holiday season. At the beginning of the study, we reminded participants that it was the holiday shopping season. Consequently, we would show them several products and have them find the best online deal (the lowest price) for each product. Participants in the high-reward condition were further told that at the end of the survey, we would show them the best price we found for each product. If the price they found was equal to or lower than our price, they would get a $0.1 bonus for the product. Participants in the low-reward condition were not told that they could earn a bonus and therefore would only receive the compensation for completing the study.

Next, we showed participants five products: a Bluetooth speaker, an electric toothbrush, a WiFi router, a hard drive, and a pair of headphones. For each product, we asked them to paste the link of the deal they found and enter the price. Before showing each product, we asked participants to indicate how much mental energy they had at that moment. To better capture changes in mental energy over time, we used the following measure (1 = “less energy than usual” and 7 = “more energy than usual”):

We would like to know how much mental energy you have at this moment. People’s mental energy fluctuates on a moment-to-moment basis. We will ask you to indicate how much mental energy you have at various times in this study. On the following scale, please indicate how much energy you feel you have AT THIS MOMENT.

We used a single-item measure because mental energy as a cognitive resource is a concrete, single-component construct (for similar measures, see Allen et al., 2014; Laran and Buechel, 2017; Cardini and Freund, 2020). Single-item measures of constructs have similar predictive validity to multiple-item measures provided (1) the construct is uni-component (e.g., mental energy) as opposed to multi-component (e.g., state and trait feelings of mental energy) and (2) the measure is of the construct (e.g., the amount of mental energy), not an attribute of the construct (e.g., the intent to use mental energy; Bergkvist and Rossiter, 2007; Diamantopoulos et al., 2012). Moreover, the single-item measure allows us to repeatedly assess mental energy in a short period of time, without introducing measurement-based rest periods that might allow mental energy to replenish (Masicampo et al., 2014; Helton and Russell, 2017).

After completing the fifth deal-finding task (i.e., the fifth product), participants saw the following message: “Congratulations! You have completed the task.” Then, they responded to the same mental energy measure as shown above. To guard against the alternative explanation that greater pay leads to more mental energy, this last measure occurred after task completion but before disclosing the amount of the bonus. To rule out the alternative explanations of felt achievement and competence (i.e., processes associated with intrinsic motivation), we also asked participants to indicate (1) the extent to which they thought completing the task felt like an achievement and (2) how effective they felt at the task, both on 7-point scales (1 = not at all and 7 = very much). Afterward, participants in the high-reward condition were shown the best deals. We compared the prices and awarded bonuses. The average bonus was $0.35 in the high-reward condition, with 98% of the participants receiving a bonus. Finally, all participants entered demographic information and were thanked for their time. The entire set of procedures and stimuli of this and all studies in the paper can be found in the Supplementary Material.

#### Results

##### Post-test of Unexpected Effort Assumption

A test was used to confirm that effort was higher than expected. An independent sample of participants (*N* = 27) engaged in the same task without mental energy measures. Participants indicated how effortful they thought the task would be on a 7-point scale (1 = not at all effortful and 7 = effortful) prior to engaging in the task (i.e., a measure of expected effort). Upon completion, participants indicated how effortful they thought the task was on the same 7-point scale (1 = not at all effort and 7 = effortful; i.e., a measure of actual effort). As expected, actual effort (*M* = 5.63, *SD* = 1.39) was higher than expected effort [*M* = 4.96, *SD* = 1.48; *F*(1, 26) = 10.40, *p* = 0.003, $\omega_{p}^{2}$ = 0.251].

##### Analysis of Mental Energy

**Figure 3:**
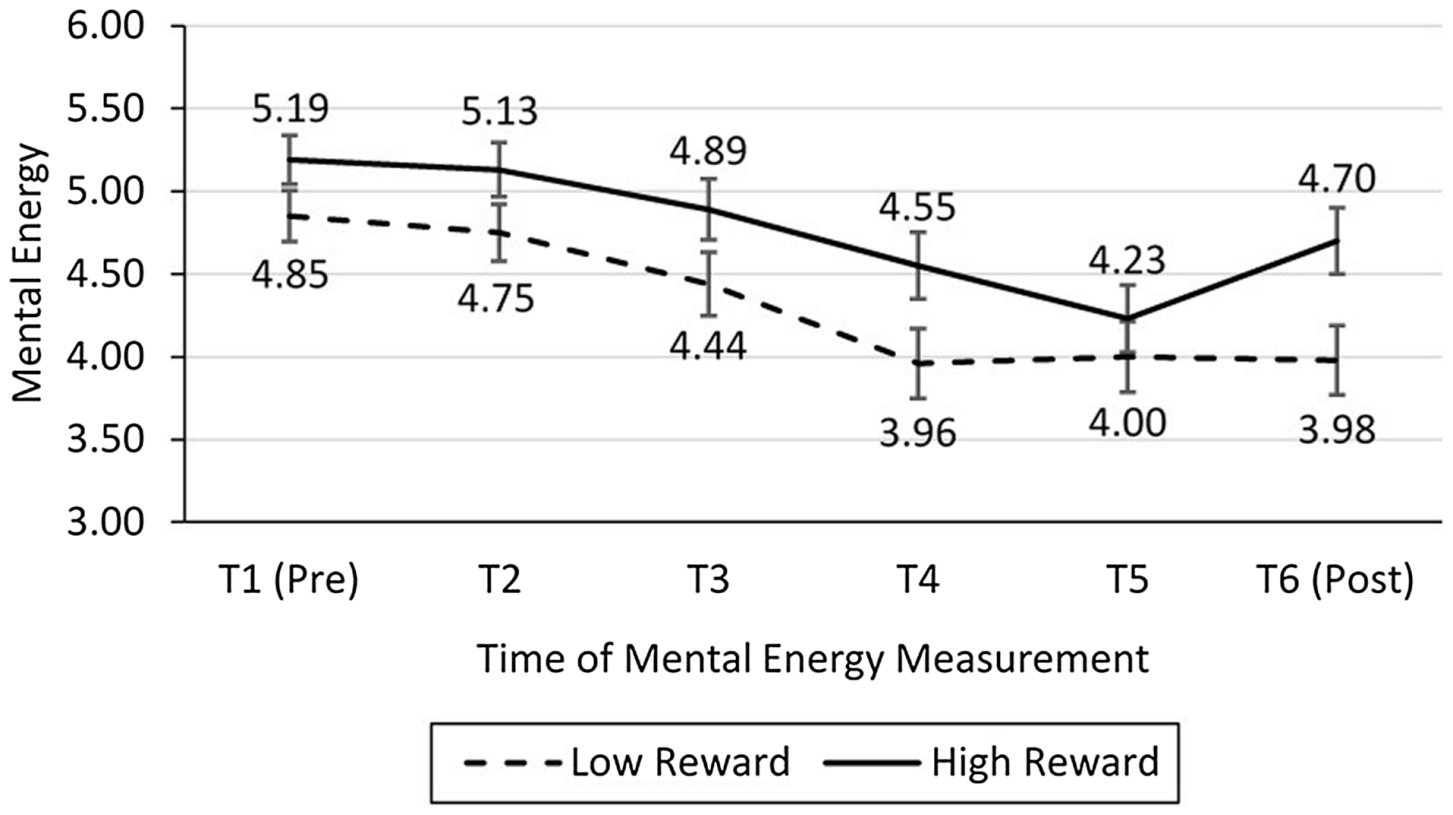
Mental energy at different points in the task (study 1).

Mental energy was measured six times in total. The average ratings across times and conditions are shown in Figure 3. T1 through T5 indicate the amount of mental energy reported before participants started to search for the first, second, … fifth product, respectively, and T6 was the amount of mental energy reported upon task completion. To examine mental energy replenishment pre- vs. post-task completion, we used a repeated measures ANOVA with reward value (low vs. high) as a between-subjects factor and time (T5: pre-completion vs. T6: post-completion) as a within-subjects factor.

Mental energy replenishment was measured as the difference in mental energy at time T5 and time T6. This within-subject measure was better than analyzing mental energy at T6, because mental energy at T6 could not adjust for difference in mental energy at T5 (i.e., mental energy at T6 is not a measure of replenishment). Consistent with hypothesis 1, there was a significant interaction between time (T5 vs. T6) and reward value [*F*(1, 99) = 6.71, *p* = 0.011, $\omega_{p}^{2}$ = 0.054; see Figure 3]. Follow-up pairwise comparisons revealed that participants in the high-reward condition exhibited mental energy replenishment [*M*_T5_ = 4.23, *SD* = 1.57; *M*_T6_ = 4.70, *SD* = 1.37; *F*(1, 99) = 12.95, *p* = 0.001, $\omega_{p}^{2}$ = 0.106], whereas participants in the low-reward condition exhibited no mental energy replenishment [*M*_T5_ = 4.00, *SD* = 1.37; *M*_T6_ = 3.98, *SD* = 1.54; *F*(1, 99) = 0.02, *p* = 0.880].

A final set of analyses confirmed that mental energy generated as a consequence of extrinsic task completion did not depend on intrinsic task mediators like felt achievement or competence. The effect of reward value on felt achievement and competence was not significant [felt achievement: *M*_low_ = 4.58, *SD*_low_ = 1.84; *M*_high_ = 5.04, *SD*_high_ = 1.51; *F*(1, 99) = 1.86, *p* = 0.176; competence: *M*_low_ = 4.92, *SD*_low_ = 1.69; *M*_high_ = 5.43, *SD*_high_ = 1.46; *F*(1, 99) = 2.73, *p* = 0.102]. A follow-up test showed the interaction between time (T6 – T5) and reward on mental energy remained significant after controlling for felt achievement and competence [*F*(1, 97) = 5.50, *p* = 0.021, $\omega_{p}^{2}$ = 0.043]. The correlations between mental energy replenishment (i.e., T6 – T5 difference) and achievement or competence were not significant in the high-reward condition (achievement: *r* = 0.028, *p* = 0.841; competence: *r* = −0.054, *p* = 0.699) or low-reward condition (achievement: *r* = 0.160, *p* = 0.276; competence: *r* = 0.257, *p* = 0.078). The lack of a significant correlation in the high-reward condition is additional evidence that felt achievement and competence were not responsible for the increase in mental energy.

##### Replication With a Low-Reward Condition

One may argue that participants in low-reward condition received no reward, which is not equivalent to low reward. To address this concern, we reran study 1 (*N* = 118) using a low-reward condition, where participants received a $0.01 bonus for each best deal they found. The amount of bonus in the high-reward condition was $0.10. The results replicated the main findings of study 1: The significant interaction between time (T5 vs. T6) and reward value was significant [*F*(1, 116) = 4.38, *p* = 0.039, $\omega_{p}^{2}$ = 0.028] and participants in the high-reward condition exhibited mental energy replenishment [*M*_T5_ = 4.33, *SD* = 1.42; *M*_T6_ = 4.62, *SD* = 1.41; *F*(1, 116) = 7.71, *p* = 0.006, $\omega_{p}^{2}$ = 0.054] while participants in the low-reward condition did not [*M*_T5_ = 4.32, *SD* = 1.66; *M*_T6_ = 4.30, *SD* = 1.74; *F*(1, 116) = 0.03, *p* = 0.873; see the Supplementary Material for a full analysis]. Thus, using a low-reward condition instead of a no reward condition does not change our conclusions.

#### Discussion

Study 1 provides evidence that mental energy is replenished at task completion when the reward value is high, but not when the reward value is low. Further, it rules out the possibility that the influence of reward value on mental energy replenishment is due to intrinsic motivation mediators like feelings of achievement or competence. This null effect was anticipated because mental energy replenishment after an extrinsically motivated task should not be sensitive to drivers of intrinsic motivation.

### Study 2

Study 2 demonstrates the behavioral implications of post-task mental energy replenishment. Specifically, study 2 replicates the findings of study 1 using a behavioral measure (e.g., task persistence) instead of a self-report of mental energy. A self-report of mental energy was not included in this study in order to avoid contamination across measures (i.e., directing attention to one’s mental energy level may lead to demand artifacts on a behavioral measure; performance on a behavioral measure may lead to inferences about one’s mental energy level).

Study 2 included an additional factor meant to address the possibility that mental energy replenishment is a consequence of intrinsic motivation contaminating an extrinsically motivated task. Energy management in intrinsic motivation occurs in-task (Csikszentmihalyi and LeFevre, 1989; Deci and Ryan, 2000). That is, if a task is enjoyable or engaging, mental energy can be allocated in-task so that the behavior is sustained. To address this possibility, task completion was manipulated across conditions. Using the same procedure as in study 1, participants were either told the task was completed or not after finishing the fifth part of the procedure. If intrinsic motivation was contaminating the extrinsically motivated task, this manipulation should not matter. If mental energy replenishment is a function of effort and reward at task completion (i.e., a post-task event), then there should only be mental energy replenishment in the high reward – task completion condition.

#### Method

##### Participants and Design

The experiment used a 2 (reward value: low vs. high) by 2 (completion: yes vs. no) between-subject design. An *a priori* power analysis using G*Power 3.1 (Faul et al., 2009) determined that at least 199 participants would be required to detect a small-to-medium interaction effect (*f* = 0.20) with a power of 80%. We targeted a total sample of 300 on Mechanical Turk, and 295 participants completed the study in exchange for $1.30 in compensation. Forty-eight participants did not enter any relevant links throughout the task and, therefore, were excluded, leaving 247 participants (*M*_age_ = 34.01, 61.9% male).

##### Procedure and Stimuli

Participants completed a deal-search task, as in study 1, but with five changes. First, as the study was conducted in April (non-holiday season), we removed holiday-related words and pictures from the instructions. Second, we removed all measures of mental energy. Third, task completion was manipulated after participants completed the fifth deal-finding task. Participants in the completion condition saw the following message: “Congratulations! You have completed this task.” On the next page, they read “Now we would like you to complete another task.” Participants in the no completion condition saw a page saying “loading the next item” (all participants saw the same page after they finished the first, second, third, and fourth deal-finding task). On the next page, they read “Now we would like you to switch to another task.” Fourth, the specifics of some products were changed (e.g., color and model type) due to product availability or deal availability. Finally, given the need to immediately measure task persistence, and the null effects in study 1, felt achievement and competence were not measured.

The availability of mental energy was measured using persistence on the second task (Braver, 2012). Task persistence owing to cognitive resources has been operationalized as sustained effort on unsolvable puzzles (Baumeister et al., 1998), time spent studying (Vohs et al., 2014), continued vigilance (See et al., 1995), product evaluation (Laran and Janiszewski, 2011), and discovering embedded figures (Vohs and Heatherton, 2000). In this study, participants did a “Book Evaluation Task.” Specifically, participants were told as: “On the next few pages, we would like you to evaluate some newly released books and tell us whether you will consider adding them to your reading list. On each page, we will show you the book title, author, and a synopsis. After you evaluate some books, you can choose to quit the task. You can quit the task whenever you like.” In this task, each book was presented on a separate screen and evaluated using two items: “Would you consider adding this book to your reading list?” (1 = yes or 2 = no) and “To what extent are you interested in reading this book?” (1 = not at all and 7 = very much). After evaluating each book, participants were offered the opportunity to “continue working on the task (i.e., evaluate more books)” or “quit.” The number of books each participant evaluated before quitting the task was the measure of task persistence.

#### Results

**Figure 4:**
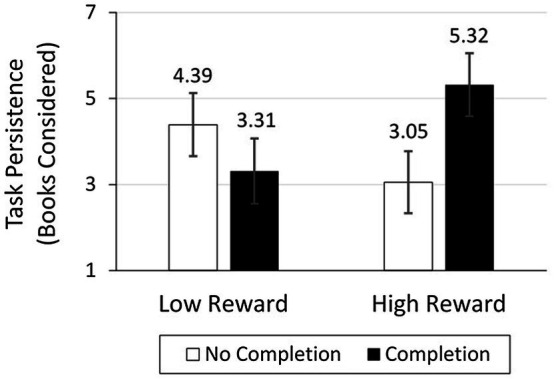
Task persistence in study 2.

Task persistence (i.e., number of books considered) was coded as the number of times each participant selected “continue working on the task (i.e., evaluate more books),” ranging from 0 to 20. There was a non-significant main effect of task reward [*F*(1, 243) = 0.21, *p* = 0.65] and completion [*F*(1, 243) = 0.66, *p* = 0.42], as well as a significant interaction between reward value and completion on task persistence [*F*(1, 243) = 5.16, *p* = 0.024; $\omega_{p}^{2}$ = 0.017; see Figure 4]. When reward value was low, participants in the completion (*M* = 3.31, *SD* = 5.24) and no completion (*M* = 4.39, *SD* = 6.28) conditions considered a similar number of books [*F*(1, 243) = 1.04, *p* = 0.31]. However, when reward value was high, participants in the completion condition (*M* = 5.32, *SD* = 6.94) considered more books than those in the no completion condition [*M* = 3.05, *SD* = 4.32; *F*(1, 243) = 4.89, *p* = 0.028; $\omega_{p}^{2}$ = 0.016].

#### Discussion

The results of study 2 provide evidence that post-task mental energy replenishment has consequences for subsequently performed behaviors. When the reward value was high, task completion increased persistence in a subsequent, unrelated task. However, when the reward value was low, task completion did not increase persistence in the subsequent task. The task completion moderator provides further evidence that mental energy replenishment is a function of the unexpected effort invested and reward accrued from an extrinsically motivated task and that mental energy replenishment does not occur in-task. Study 2 also addresses the alternative explanation that measuring mental energy makes people more sensitive to mental energy. In study 2, there were no measures of mental energy, yet the results replicated study 1. Second, it could be argued that measuring mental energy creates a demand effect on reports of mental energy in high-reward conditions. In study 2, the high reward was kept constant, and no measure of mental energy was collected to make mental energy salient, yet the consequences of mental energy replenishment were still obtained in the completion condition. This should reduce concerns about demand effects.

### Study 3

The x-axis in Figure 2 is the difference between expected effort and actual effort. We hypothesize that people replenish mental energy after completing an extrinsically motivated task only when actual effort exceeds the expected effort by a sufficient amount (see A2 vs. A1 in Figure 2). One approach to providing evidence for this prediction is to alter the expected effort associated with a task. When actual effort sufficiently exceeds expected effort (i.e., there is unexpected effort), there should be mental energy replenishment when there is a high-reward value but not when there is a low-reward value (i.e., see A2 vs. B2 in Figure 2). This result would replicate the results of studies 1 and 2. In contrast, when actual effort does not exceed expected effort because the person has been led to believe the task will be more effortful (i.e., there is no unexpected effort), there should be no mental energy replenishment in a high or low-reward value condition (i.e., see A1 vs. B1 in Figure 1). This result would illustrate that the difference between expected and actual effort, not solely the amount of actual effort, is partially responsible for post-task mental energy replenishment.

#### Method

##### Participants and Design

The experiment used a 2 (reward value: low vs. high) by 2 (expected effort: low vs. high) between-subject design. An *a priori* power analysis using G*Power 3.1 (Faul et al., 2009) determined that at least 253 participants would be required to detect an effect of *f* = 0.018 (based on results of a pretest) with a power of 80%. We targeted a total sample of 260 on Mechanical Turk, and 263 Mechanical Turk participants completed the study in exchange for financial compensation (*M*_age_ = 38.24, 51.7% male). All participants were retained.

##### Procedure and Stimuli

At the beginning of the study, participants were told that they would complete a simple task where they would be asked to identify the correct synonym for a word. The synonym had to be chosen from four alternatives. Furthermore, participants learned that the task was programmed by Freerice – a 100% non-profit Web site that supports the United Nations World Food Program, and the aim of the task was to help end world hunger – for each answer they got right, the sponsors of Freerice would donate 1 grain of rice (low-reward condition) or 50 grains of rice (high-reward condition) to the United Nations World Food Program to help reach Zero Hunger.

Then, we manipulated the expected effort of the task. Participants in the high expected effort condition learned that the words they would see in the questions were not those frequently used in everyday life and, thus, they would need to invest extra cognitive effort. This description was intended to match their actual experience during the task (i.e., there should be no unexpected effort), as the questions were indeed moderately difficult and required some effort (see the questions listed in the Supplementary Material). Participants in the low expected effort condition were not provided information about expected effort and, thus, expected effort should be significantly below actual effort (i.e., there should be unexpected effort). Then, all participants rated the extent to which they thought the task would be effortful (1 = not at all and 7 = very effortful), which served as a manipulation check of anticipated effort.

Next, all participants started working on the task. The task involved five sets of questions, five questions in each set. To remind participants of the task reward, we put a banner at the top of the page saying “Free rice” and “For each answer you get right, we donate [1/50] grains of rice through the World Food Program to help end hunger.” Upon completion of the fifth set of questions, participants saw the following message: “Congratulations! You have completed the task.” As in previous studies, we measured mental energy prior to each set of five questions as well as after participants completed all sets of questions. After completing the task, participants completed a manipulation check of task reward, where they indicated the extent to which they thought the task was “valuable,” “important,” “rewarding,” and “useful” (1 = not at all and 7 = very much; Cronbach’s alpha = 0.96). Finally, participants responded to the achievement and competence measures used in studies 1 and 2.

#### Results

##### Manipulation Checks

As expected, there was a significant main effect of the reward value manipulation on perceived reward value [*M*_high reward_ = 4.88, *M*_low reward_ = 4.37; *F*(1, 259) = 6.68, *p* = 0.010, $\omega_{p}^{2}$ = 0.021]. There was no main effect of expected effort or a reward value by expected effort interaction on perceived reward value. In addition, a two-way ANOVA revealed a significant main effect of the expected effort manipulation on anticipated effort [*M*_high expected effort_ = 5.78, *M*_low expected effort_ = 4.13; *F*(1, 259) = 92.67, *p* < 0.001, $\omega_{p}^{2}$ = 0.260]. There was no main effect of reward value or a reward value by expected effort interaction on anticipated effort. Furthermore, consistent with our theory, there was a main effect of the expected effort manipulation on the amount of mental energy reported at T1, prior to engaging in the task [*M*_high expected effort_ = 5.31 *M*_low expected effort_ = 5.04; *F*(1, 259) = 3.98, *p* = 0.047, $\omega_{p}^{2}$ = 0.011], suggesting that participants allocated more mental energy to the task after they learned that the task would be effortful. There was no main effect of reward value or interaction on mental energy at T1.

##### Pretest

It was important to confirm that the low expected effort condition, but not the high expected effort condition, resulted in unexpected effort. An independent sample of participants (*N* = 122) engaged in the same task except that the mental energy measures were removed. Participants indicated how effortful they thought the task would be on a 7-point scale (1 = not at all effortful and 7 = effortful) before engaging in the task. Upon completion, participants indicated how effortful the task was on the same 7-point scale (1 = not at all effort and 7 = effortful; i.e., a measure of actual effort). The expected effort manipulation by expected vs. actual effort dependent measure interaction was significant [*F*(1, 120) = 20.51, *p* < 0.001, $\omega_{p}^{2}$ = 0.138]. As expected, in the low expected effort condition, the ratings of actual effort (*M* = 5.10, *SD* = 1.54) were higher than those of expected effort [*M* = 4.30, *SD* = 1.49; *F*(1, 120) = 19.54, *p* < 0.001, $\omega_{p}^{2}$ = 0.132]. In the high expected effort condition, however, actual effort (*M* = 5.38, *SD* = 1.55) was lower than expected effort [*M* = 5.74, *SD* = 1.32; *F*(1, 119) = 3.94, *p* = 0.049, $\omega_{p}^{2}$ = 0.024].

##### Mental Energy

**Figure 5:**
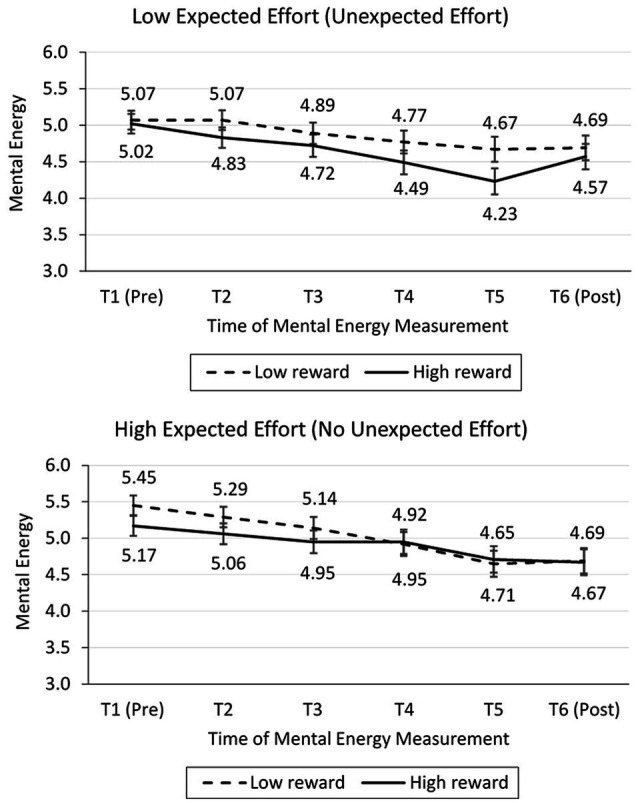
Results of study 3.

Mental energy was measured six times. Consistent with our predictions, there was a three-way interaction of reward value, expected effort, and time [E5: pre-completion vs. E6: post-completion; *F*(1, 259) = 4.49, *p* = 0.035, $\omega_{p}^{2}$ = 0.013]. Further, there was a two-way interaction between reward value and time in the low expected effort condition [*F*(1, 259) = 5.55, *p* = 0.019, $\omega_{p}^{2}$ = 0.017; see Figure 5]. Specifically, there was mental energy replenishment in the high-reward value condition [*M*_T5_ = 4.23, *SD* = 1.53, *M*_T6_ = 4.57, *SD* = 1.46; *F*(1, 259) = 11.67, *p* = 0.001, $\omega_{p}^{2}$ = 0.039], but not the low-reward value condition [*M*_T5_ = 4.67, *SD* = 1.25, *M*_T6_ = 4.69, *SD* = 1.21; *F*(1, 259) = 0.02, *p* = 0.881]. In the high expected effort condition, there was no interaction between reward value and time [*F*(1, 259) = 0.44, *p* = 0.51] and no main effect of time [*F*(1, 259) = 0.00, *p* = 0.992; see Figure 5] on mental energy replenishment.

##### Additional Analyses Using Felt Achievement and Competence

Similar to study 1, we anticipated that the mediators of intrinsic motivation would not explain mental energy replenishment for an extrinsically motivated task. Two-way ANOVAs revealed a main effect of reward value on felt achievement [*M*_high value_ = 5.03, *M*_low value_ = 4.41; *F*(1, 259) = 9.40, *p* = 0.002, $\omega_{p}^{2}$ = 0.031] and a marginally significant main effect of reward value on competence [*M*_high value_ = 4.77, *M*_low value_ = 4.43; *F*(1, 259) = 3.47, *p* = 0.063, $\omega_{p}^{2}$ = 0.009]. In the low expected effort condition, the correlation between mental energy replenishment (T6 – T5) and felt achievement or competence was not significant in the low-reward condition (achievement: *r* = 0.047, *p* = 0.698; competence: *r* = −0.065, *p* = 0.594) or the high-reward condition (achievement: *r* = 0.123, *p* = 0.330; competence: *r* = −0.009, *p* = 0.945). In the high expected effort condition, there was a marginally significant correlation between mental energy replenishment (T6 – T5) and felt achievement (*r* = 0.151, *p* = 0.090) while the correlation between mental energy replenishment (T6 – T5 and) competence was not significant (*r* = 0.078, *p* = 0.383). The lack of a significant correlation in the low expected effort – high-reward condition suggests that felt achievement and competence were not responsible for the replenishment of mental energy.

#### Discussion

Study 3 provides evidence that unexpected effort (i.e., the need to replenish), not actual effort, is responsible for post-task mental energy replenishment. When actual effort exceeded expected effort, and there was a high reward (i.e., a favorable cost-benefit trade-off), mental energy was replenished. When actual effort was less than the expected effort, and there was a high reward, there was no mental energy replenishment.

Study 3 helps rule out potential alternative explanations. For example, it could be argued that high rewards generate affect and this positive affect increases mental energy. This prediction is inconsistent with the results of study 3 because the low and high expected effort conditions both provided a high reward, but only the low expected effort condition resulted in increased mental energy. Similarly, it could be argued that high rewards encourage arousal, excitement, or anticipation that increase mental energy. Again, the interaction effect makes this unlikely. Study 4 creates a quadratic effect in the high-reward condition and, thus, provides additional evidence against these explanations.

### Study 4

The x-axis in Figure 2 is the difference between actual effort and expected effort. In study 4, we held expected effort constant, while manipulating actual effort and the size of the reward. When the reward was high, we expected to show the inverted-U pattern illustrated by A1, A2, and A3 in Figure 2. When the reward was low, we expected to show the flat pattern illustrated by B1, B2, and B3 in Figure 2.

#### Method

##### Participants and Design

The study was preregistered on AsPredicted.org.^1^ An *a priori* power analysis using G*Power 3.1 (Faul et al., 2009) suggested a minimal sample size of 351 to detect an interaction effect of *f* = 0.15 (based on results of a pretest) with a power of 80%. We aimed to recruit 400 participants on Mechanical Turk, and a total of 401 participants completed the study in exchange for financial compensation. Eleven participants did not provide relevant responses and were therefore excluded from analyses using preregistered exclusion criteria, leaving a final sample of 390 participants (*M*_age_ = 37.00, 38.2% male). The experiment used a 2 (reward value: low vs. high) by 3 (actual effort: low vs. moderate vs. high) between-subject design.

##### Procedure

At the beginning of the study, participants were told that they would see information tags for electronic products and their task was to transcribe the product information into digital text. The transcription task required participants to transcribe five product descriptions.

**Figure 6:**
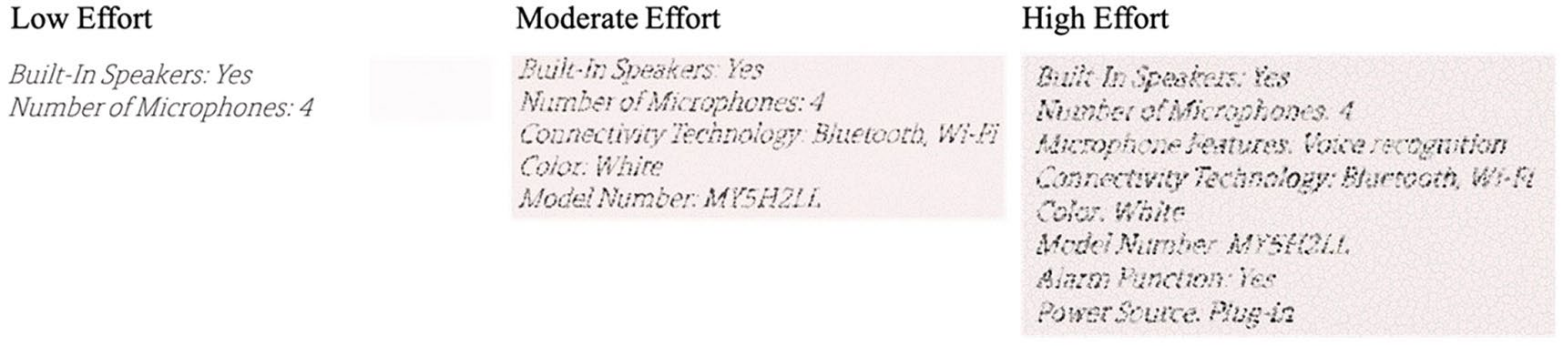
Stimuli used in study 4.

To manipulate reward value, we told participants that they would either receive an extra 1 cent (low-reward condition) or an extra 5 cents (high-reward condition) for each information tag they accurately transcribed. Next, participants started working on the task. All participants transcribed a set of five information tags, with text length and blurriness varying across the three effort conditions. In the low effort condition, each information tag had two product attributes on it (about 40–50 characters) and text was clear (see Figure 6 and the Supplementary Material). In the moderate effort condition, each information tag had five product attributes (about 100–120 characters) and the text was degraded a little, so participants had to spend more effort recognizing the text and typing it out. In the high effort condition, each information included eight product attributes (about 160–180 characters) and text was degraded to a greater extent (but still recognizable). A measure of mental energy was taken prior to starting the task (T1) and after each of the five sets of transcriptions (T2 – T6). After transcribing the fifth tag, just prior to the T6 measure of mental energy, participants saw “Congratulations! You have completed the task.”

After completing the task, participants responded to several follow-up questions. First, as a measure of positive affect, they reported how happy they were at the moment. Next, participants responded to the achievement and competence measures used in studies 1 and 3. Finally, as a manipulation check of reward value, we measured how rewarding participants thought the extra payment for accurately transcribing the information tag was on a 7-point scale (1 = not at all and 7 = very much).

#### Results

##### Pretest

To assess if the actual task effort manipulation was successful, and if actual effort was higher than expected effort, a pretest had participants (*N* = 192) engage in the same procedure as in the main experiment, except that the information about the extra reward payment and the mental energy measures were removed. Participants indicated expected effort prior to engaging in the task using a 7-point scale (1 = not at all effortful and 7 = effortful). Upon completing the task, participants indicated actual effort using a 7-point scale (1 = not at all effort and 7 = effortful).

As predicted, the interaction between the actual effort manipulation and the difference between expected and actual effort (repeated measure) was significant [*F*(2, 189) = 12.73, *p* < 0.001, *ω*^2^ = 0.116]. Actual effort was lower than expected effort in the low effort condition [*M*_expected_ = 4.77, *SD* = 1.48, *M*_actual_ = 4.21, *SD* = 1.62; *F*(1, 189) = 8.48, *p* = 0.004, *ω*^2^ = 0.038], but higher than expected effort in the moderate effort condition [*M*_expected_ = 4.72, *SD* = 1.70, *M*_actual_ = 5.14, *SD* = 1.96; *F*(1, 189) = 4.59, *p* = 0.034, *ω*^2^ = 0.018] and the high effort condition [*M*_expected_ = 4.92, *SD* = 1.30, *M*_actual_ = 5.79, *SD* = 1.60; *F*(1, 189) = 14.65, *p* < 0.001, *ω*^2^ = 0.067].

##### Manipulation Check

A two-way ANOVA revealed a main effect of the reward value manipulation on perceived reward value [*M*_high value_ = 5.14, *M*_low value_ = 4.18; *F*(1, 384) = 22.59, *p* < 0.001, $\omega_{p}^{2}$ = 0.053]. There was also a marginally significant effect of actual effort on perceived reward value [*M*_low effort_ = 4.84, *M*_moderate effort_ = 4.78, *M*_high effort_ = 4.32; *F*(2, 384) = 2.44, *p* = 0.089, $\omega_{p}^{2}$ = 0.007]. However, the interaction between reward value and actual effort was not significant (*F* < 1).

##### Mental Energy

**Figure 7:**
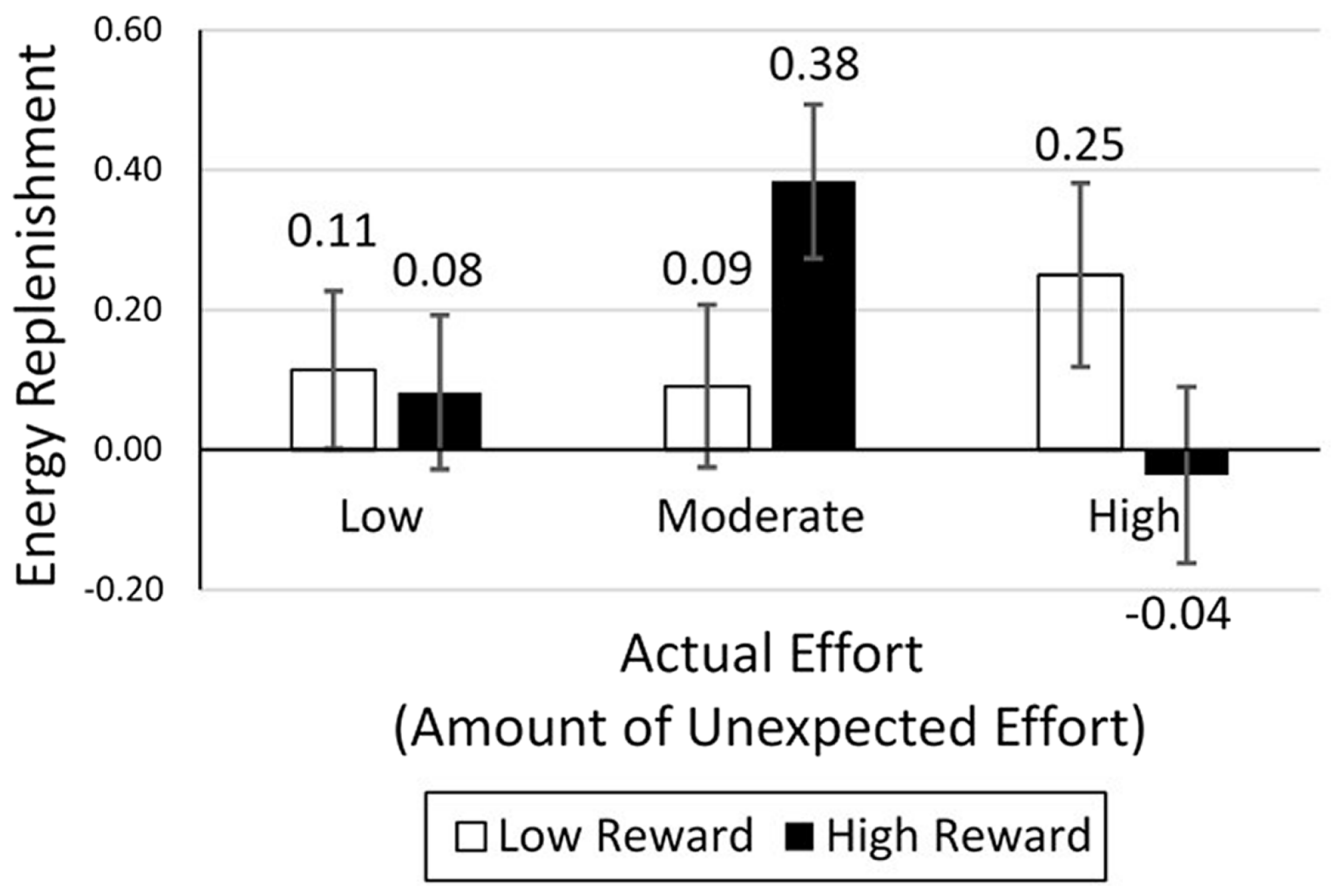
Mental energy replenishment in study 4.

As in study 1 and study 3, mental energy was measured six times. As preregistered, we first computed the amount of mental energy replenishment by subtracting pre-completion mental energy (T5) from post-completion mental energy (T6). Then, we created the planned quadratic contrast for the actual effort manipulation (e.g., low = −1, moderate = 2, and high = −1), which has a single degree of freedom associated with it. We used this planned contrast in the mental energy analyses. The means for the T6 – T5 difference (energy replenishment) are reported in Figure 7. The means for T1 through T6, for all conditions, are reported in the Supplementary Material.

The analysis was conducted using the preregistered plan. There was an interaction between reward value and actual effort on mental energy replenishment [*F*(1, 384) = 5.09, *p* = 0.025, $\omega_{p}^{2}$ = 0.010]. In the high-reward condition, there was a significant effect of effort on mental energy replenishment [*F*(1, 384) = 6.77, *p* = 0.010, $\omega_{p}^{2}$ = 0.015]. More importantly, planned contrasts revealed that mental energy replenishment in the moderate effort condition (*M* = 0.38, *SD* = 0.97) was higher than in the low effort condition [*M* = 0.08, *SD* = 0.91; *t*(384) = 1.93, one-tailed test, *p* = 0.027, $\omega_{p}^{2}$ = 0.007] and the high effort condition [*M* = −0.04, *SD* = 0.87; *t*(384) = 2.50, one-tailed test, *p* = 0.006, $\omega_{p}^{2}$ = 0.013]. In the low-reward condition, there was no effect of effort [*F*(1, 384) = 0.398, *p* = 0.528]. Planned contrasts showed no difference in mental energy replenishment between the moderate effort (*M* = 0.09, *SD* = 1.03) and low effort (*M* = 0.11, *SD* = 0.65) condition [*t*(384) = −0.14, *p* = 0.885] or the moderate effort (*M* = 0.09, *SD* = 1.03) and high effort (*M* = 0.25, *SD* = 1.20) condition [*t*(384) = −0.91, *p* = 0.363]. These results are consistent with Figure 2.

##### Positive Affect, Felt Achievement, and Competence

Two-way ANOVAs revealed a main effect of effort on positive affect [*M*_low effort_ = 4.99, *M*_moderate effort_ = 4.96, *M*_high effort_ = 4.45; *F*(2, 384) = 4.24, *p* = 0.015, $\omega_{p}^{2}$ = 0.017] and a marginally significant main effect of effort on competence [*M*_low effort_ = 5.97, *M*_moderate effort_ = 5.84, *M*_high effort_ = 5.58; *F*(2, 384) = 2.78, *p* = 0.063, $\omega_{p}^{2}$ = 0.009]. Tukey *post-hoc* tests revealed that positive affect was lower in the high effort condition than in the low effort condition (*p* = 0.019, *d* = 0.34) and the moderate effort condition (*p* = 0.030, *d* = 0.32). Competence was lower in the high effort condition than that in the low effort condition (*p* = 0.049, *d* = 0.30). The lower ratings in the high effort condition could be driven by the fact that the task was much more effortful than what participants expected and thus was frustrating and made participants feel less competent. No other main effects or interactions were significant. In summary, the patterns of positive affect and competence cannot explain the interaction of reward value and effort on replenishment nor the quadratic effect we observed in the high-reward condition.

#### Discussion

Study 4 provides additional evidence that the amount of unexpected effort determines the amount of mental energy replenishment, provided the reward for engaging in the behavior is high. When unexpected effort was moderate, and the cost-benefit trade-off was favorable (i.e., the reward is high), there was mental energy replenishment. When there was no unexpected effort, there was no need to replenish mental energy. When unexpected effort was large, the cost-benefit trade-off was unfavorable and there was no mental energy replenishment. We contend that the lack of mental energy replenishment reflects an automatic strategy for using the mental energy deficit as a signal to take corrective action.

## General Discussion

We provide evidence for mental energy replenishment in an extrinsically motivated task. We show that when the effort expended in an extrinsically motivated task is significantly more than expected, and the actual the cost-benefit trade-off is favorable, mental energy is replenished at the completion of the task (see Figure 2). If the extrinsically motivated task does not provide a sufficient reward (studies 1–4; Figure 2 B1, B2, and B3), or if unexpected effort is minimal (studies 3 and 4; Figure 2 A1) or extreme (study 4; Figure 2 A3), there will be marginal mental energy replenishment. Mental energy replenishment is a useful adjustment mechanism, so that energy deficits resulting from a conservative mental energy allocation system do not result in compromised performance on future tasks. Mental energy replenishment for extrinsically motivated tasks can also be useful when there is cumulative learning over time and it is difficult to anticipate the mental energy requirements of activities in the learning process. Learning is more likely to persist when rewarding learning is accompanied by mental energy replenishment.

### Implications for the Management of Mental Energy Resources

We believe that mental energy replenishment subsequent to a rewarding, extrinsically motivated task is necessary for sustained, effective cognitive behavior. To understand why, consider what we know about mental energy management. First, people have a baseline level of mental energy (Shulmana et al., 2009). This baseline level of mental energy supports consciousness and cognition. Second, people can increase the baseline level of mental energy. Pre-task allocations of mental energy are sensitive to factors, such as anticipated demands and rewards of the upcoming task (Beedie and Lane, 2012; Kruglanski et al., 2012; Shenhav et al., 2013), so that people can engage in beneficial cognitive behaviors. Third, there are incentives to be conservative with mental energy allocation as mental effort is costly, both absolutely and from an opportunity cost perspective (Boksem and Tops, 2008; Kurzban et al., 2013; Goldfarb and Henik, 2014). This leads to mental energy deficits, especially when energy use exceeds energy allocation on consecutive tasks (Kanfer et al., 1994). Fourth, pre-task allocations of mental energy cannot address a cumulative deficit. Thus, an energy replenishment function is conceptually consistent with the prior literature on mental energy use. Replenishment is a necessary part of an effective mental energy management system.

Perhaps, the most curious characteristic of the mental energy replenishment system is its sensitivity to the cost-benefit trade-off of a completed task. As shown in Figure 2, there is no mental energy replenishment when the cost-benefit trade-off is unfavorable (i.e., A3, B2, and B3 in Figure 2). We argue that this is adaptive because a replenishment system that is insensitive to the efficiency of energy investment would continue to replenish mental energy, and prepare for the next task, regardless of task difficulty. If a person is in an unfamiliar environment that creates mental energy deficits, then it would be beneficial for the person to experience low energy (i.e., baseline mental effort should continue to drop) and disengage from the environment/task if the rewards do not justify the actual cognitive effort (Kanfer et al., 1994). In fact, this is exactly what happens when people experience low energy (Kurzban et al., 2013; Cardini and Freund, 2020). They disengage from the environment/task. When rewards are insufficient given the effort, there is no post-task energy replenishment and the resulting lack of energy leads to disengagement from subsequent tasks (Hopstaken et al., 2015).

In this sense, our research also speaks to the literature on the adaptive nature of mental energy levels (e.g., Kool et al., 2010; Kurniawan et al., 2011; Kurzban et al., 2013; Botvinick and Braver, 2015). A growing literature suggests that a lack of mental energy could be an adaptive signal of the need to abandon or change the course of the ongoing behavior, because the current behavioral strategy may not be the most appropriate (Boksem and Tops, 2008). Our research suggests that the replenishment of mental energy may well be a signal to continue the pursuit of other rewarding, extrinsically motivated tasks, rather than shifting task priorities away from “have-to” goals (e.g., work tasks) to “want-to” goals (e.g., leisure tasks; Robinson et al., 2010; Inzlicht and Schmeichel, 2012; Inzlicht et al., 2014).

### Limitations

The research procedures used in this paper have limitations. First, the procedure relies on a single-item measure of mental energy. Cognitive resource levels are more commonly assessed by measuring repeated performance on a task, with decrements in performance indicating decreased cognitive resource levels (See et al., 1995). Our single-item measure likely reflects an indirect assessment of cognitive resource levels (e.g., a perception of changes in the difficulty of executing a repeated task), since a meta-cognitive assessment of actual cognitive resource levels is difficult.

Second, we used tasks for which a person can make fairly accurate assessments of cognitive resource demands, so that mental energy can be allocated pre-task and deficits can occur post-task. Assessments of resource demands are only relevant in situations where tasks demands are predictable. There are many situations where tasks are ambiguous owing to the complexity of the problem (Dörner and Funke, 2017). The problem solving space may be ambiguous, complex, uncertain, and volatile. In these types of domains, it is difficult to anticipate rewards associated with an outcome, anticipate mental energy requirements, allocate pre-task resources, and determine stopping points (e.g., task completion). Thus, it remains to be seen whether our results generalize to management of mental energy beyond the performance of simple, common tasks.

Third, our behavior-based evidence for mental energy replenishment is limited to a single study (study 2) that measures performance on an unfixed task (i.e., people perform a self-paced book-review task for as long as they like). The performance of unfixed tasks can be sensitive to any antecedent that increases the availability of mental energy. In addition to mental energy replenishment, our favored explanation, it is also possible that motivational factors influenced the supply of energy. The successful completion of an initial task (e.g., rewarding shopping task) could activate the trait grit (the motivation to complete tasks) and encourage a larger investment of energy in a subsequent task (e.g., reviewing books; Duckworth et al., 2007).

### Conclusion

People need mental energy to complete cognitive tasks. In this research, we document a novel source of mental energy. Mental energy can be generated at the completion of an extrinsically motivated task, assuming the task created a mental energy deficit and the cost-benefit trade-off for the task was favorable. These results reflect the idea that engaging in high-reward activities can be self-sustaining, even if the activities are more difficult than expected. Future research can investigate additional factors that interact with unexpected effort and reward value to generate mental energy at task completion. An investigation of these issues will provide better insight into how people replenish mental energy throughout their day and, consequently, sustain productive behavior.

## Data Availability Statement

The raw data supporting the conclusions of this article will be made available by the authors, without undue reservation.

## Ethics Statement

The studies involving human participants were reviewed and approved by the University of Florida Institutional Review Boards. The patients/participants provided their written informed consent to participate in this study.

## Author Contributions

XW and CJ contributed to the study designs. XW collected and analyzed the data under the guidance of CJ. CJ drafted the manuscript. All authors contributed to conception of the studies and manuscript revision and approved the final manuscript for submission.

## Conflict of Interest

The authors declare that the research was conducted in the absence of any commercial or financial relationships that could be construed as a potential conflict of interest.

## Publisher’s Note

All claims expressed in this article are solely those of the authors and do not necessarily represent those of their affiliated organizations, or those of the publisher, the editors and the reviewers. Any product that may be evaluated in this article, or claim that may be made by its manufacturer, is not guaranteed or endorsed by the publisher.

## Footnotes

^1^ https://aspredicted.org/blind.php?x=xf5pv8

## References

[ref1] Allen P. M. Edwards J. A. Snyder F. J. Makinson K. A. Hamby D. M. (2014). The effect of cognitive load on decision making with graphically displayed uncertainty information. Risk Anal. 34, 1495–1505. 10.1111/risa.12161, PMID: 24354944 PMC4063894

[ref2] Baumeister R. F. Bratslavsky E. Muraven M. Tice D. M. (1998). Ego depletion: is the active self a limited resource? J. Pers. Soc. Psychol. 74, 1252–1265. 10.1037/0022-3514.74.5.1252, PMID: 9599441

[ref3] Beedie C. J. Lane A. M. (2012). The role of glucose in self-control: another look at the evidence and an alternative conceptualization. Personal. Soc. Psychol. Rev. 16, 143–153. 10.1177/1088868311419817 21896791

[ref4] Bergkvist L. Rossiter J. R. (2007). The predictive validity of multiple-item versus single-item measures of the same constructs. J. Mark. Res. 44, 175–184. 10.1509/jmkr.44.2.175

[ref5] Bettman J. R. Luce M. F. Payne J. W. (1998). Constructive consumer choice processes. J. Consum. Res. 25, 187–217. 10.1086/209535

[ref6] Bindra D. (1974). A motivational view of learning, performance, and behavioral modification. Psychol. Rev. 81, 199–213. 10.1037/h0036330, PMID: 4424766

[ref7] Bodenhausen G. V. Lichtenstein M. (1987). Social stereotypes and information-processing strategies: the impact of task complexity. J. Pers. Soc. Psychol. 52, 871–880. 10.1037/0022-3514.52.5.871, PMID: 3585699

[ref8] Boksem M. A. S. Tops M. (2008). Mental fatigue: costs and benefits. Brain Res. Rev. 59, 125–139. 10.1016/j.brainresrev.2008.07.001, PMID: 18652844

[ref9] Bolles R. C. (1972). Reinforcement, expectancy, and learning. Psychol. Rev. 29, 394–409. 10.1037/h0033120

[ref10] Boolani A. Manierre M. (2019). An exploratory multivariate study examining correlates of trait mental and physical fatigue and energy. Fatigue Biomed. Health Behav. 7, 29–40. 10.1080/21641846.2019.1573790

[ref11] Boolani A. Ryan J. Vo T. Wong B. Banerjee N. K. Banerjee S.. (2020). Do changes in mental energy and fatigue impact functional assessments associated with fall risks? An exploratory study using machine learning. Phys. Occup. Ther. Geriatr. 38, 283–301. 10.1080/02703181.2020.1748788

[ref12] Botvinick M. Braver T. (2015). Motivation and cognitive control: from behavior to neural mechanism. Annu. Rev. Psychol. 66, 83–113. 10.1146/annurev-psych-010814-015044, PMID: 25251491

[ref13] Braver T. S. (2012). The variable nature of cognitive control: a dual mechanisms framework. Trends Cogn. Sci. 16, 106–113. 10.1016/j.tics.2011.12.010, PMID: 22245618 PMC3289517

[ref14] Brehm J. W. Self E. A. (1989). The intensity of motivation. Annu. Rev. Psychol. 40, 109–131. 10.1146/annurev.ps.40.020189.000545, PMID: 2648973

[ref15] Brehm J. W. Wright R. A. Solomon S. Silka L. Greenberg J. (1983). Perceived difficulty, energization, and the magnitude of goal valence. J. Exp. Soc. Psychol. 19, 21–48. 10.1016/0022-1031(83)90003-3

[ref16] Broadbent D. E. (1979). The society’s lecture 1979: is a fatigue test possible? Ergonomics 22, 1277–1290. 10.1080/00140137908924702 540644

[ref17] Campbell D. J. (1988). Task complexity: a review and analysis. Acad. Manag. Rev. 13, 40–52. 10.5465/amr.1988.4306775

[ref18] Cardini B. B. Freund A. M. (2020). More or less energy with age? A motivational life-span perspective on subjective energy, exhaustion, and opportunity costs. Psychol. Aging 35, 369–384. 10.1037/pag0000445, PMID: 32077733

[ref19] Carver C. S. Scheier M. F. (1981). The self-attention-induced feedback loop and social facilitation. J. Exp. Soc. Psychol. 17, 545–568. 10.1016/0022-1031(81)90039-1

[ref20] Carver C. S. White T. L. (1994). Behavioral inhibition, behavioral activation, and affective responses to impending reward and punishment: the BIS/BAS scales. J. Pers. Soc. Psychol. 67, 319–333. 10.1037/0022-3514.67.2.319

[ref21] Csikszentmihalyi M. LeFevre J. (1989). Optimal experience in work and leisure. J. Pers. Soc. Psychol. 56, 815–822. 10.1037/0022-3514.56.5.815, PMID: 2724069

[ref22] Deci E. L. Ryan R. M. (2000). The “what” and “why” of goal pursuit: human needs and the self-determination of behavior. Psychol. Inq. 11, 227–268. 10.1207/S15327965PLI1104_01

[ref23] Diamantopoulos A. Sarstedt M. Fuchs C. Wilczynski P. Kaiser S. (2012). Guidelines for choosing between multi-item and single-item scales for construct measurement: a predictive validity perspective. J. Acad. Mark. Sci. 40, 434–449. 10.1007/s11747-011-0300-3

[ref24] Dörner D. Funke J. (2017). Complex problem solving: what it is and what it is not. Front. Psychol. 8:1153. 10.3389/fpsyg.2017.01153 28744242 PMC5504467

[ref25] Duckworth A. L. Peterson C. Matthews M. D. Kelly D. R. (2007). Grit: perseverance and passion for long-term goals. J. Pers. Soc. Psychol. 92, 1087–1101. 10.1037/0022-3514.92.6.1087, PMID: 17547490

[ref26] Dunn T. L. Lutes D. J. Risko E. F. (2016). Metacognitive evaluation in the avoidance of demand. J. Exp. Psychol. Hum. Percept. Perform. 42, 1372–1387. 10.1037/xhp0000236, PMID: 27123679

[ref27] Evans J. S. B. T. (2008). Dual-processing accounts of reasoning, judgment and social cognition. Annu. Rev. Psychol. 59, 255–278. 10.1146/annurev.psych.59.103006.093629, PMID: 18154502

[ref28] Evans D. R. Boggero I. A. Segerstrom S. C. (2016). The nature of self-regulatory fatigue and “ego depletion” lessons from physical fatigue. Personal. Soc. Psychol. Rev. 20, 291–310. 10.1177/1088868315597841, PMID: 26228914 PMC4788579

[ref29] Faul F. Erdfelder E. Buchner A. Lang A. G. (2009). Statistical power analyses using G*Power 3.1: tests for correlation and regression analyses. Behav. Res. Methods 41, 1149–1160. 10.3758/BRM.41.4.1149, PMID: 19897823

[ref30] Goldfarb L. Henik A. (2014). Is the brain a resource-cheapskate? Front. Hum. Neurosci. 8:857. 10.3389/fnhum.2014.00857, PMID: 25386130 PMC4208413

[ref31] Gray J. A. (1970). The psychophysiological basis of introversion-extroversion. Behav. Res. Ther. 8, 249–266. 10.1016/0005-7967(70)90069-0, PMID: 5470377

[ref32] Greenwald A. G. Leavitt C. (1984). Audience involvement in advertising: four levels. J. Consum. Res. 11, 581–592. 10.1086/208994

[ref33] Helton W. S. Russell P. N. (2017). Rest is still best: the role of the qualitative and quantitative load of interruptions on vigilance. Hum. Factors 59, 91–100. 10.1177/0018720816683509, PMID: 28146674

[ref34] Heuchert J. P. McNair D. M. (2012). Profile of Mood States. 2nd *Edn*. Toronto: Multi-Health Systems Inc.

[ref35] Hockey G. R. J. (1993). “Cognitive-energetical control mechanisms in the management of work demands and psychological health,” in Attention: Selection, Awareness, and Control: A Tribute to Donald Broadbent. eds. Baddeley A. D. Weiskrantz L. (Clarendon Press/Oxford University Press), Oxford, 328–345.

[ref36] Hopstaken J. F. van der Linden D. Bakker A. B. Kompier M. A. J. (2015). A multifaceted investigation of the link between mental fatigue and task disengagement. Psychophysiology 52, 305–315. 10.1111/psyp.12339, PMID: 25263028

[ref37] Hull C. L. (1943). Principles of Behavior: An Introduction to Behavior Theory. Oxford: Appleton-Century.

[ref38] Inzlicht M. Bartholow B. D. Hirsh J. B. (2015). Emotional foundations of cognitive control. Trends Cogn. Sci. 19, 126–132. 10.1016/j.tics.2015.01.004, PMID: 25659515 PMC4348332

[ref39] Inzlicht M. Schmeichel B. J. (2012). What is ego depletion? Toward a mechanistic revision of the resource model of self-control. Perspect. Psychol. Sci. 7, 450–463. 10.1177/1745691612454134, PMID: 26168503

[ref40] Inzlicht M. Schmeichel B. J. Macrae C. N. (2014). Why self-control seems (but may not be) limited. Trends Cogn. Sci. 18, 127–133. 10.1016/j.tics.2013.12.009, PMID: 24439530

[ref41] Inzlicht M. Werner K. M. Briskin J. L. Roberts B. W. (2021). Integrating models of self-regulation. Annu. Rev. Psychol. 72, 319–345. 10.1146/annurev-psych-061020-105721, PMID: 33017559

[ref42] James W. (1907). The energies of men. Science 25, 321–332. 10.1126/science.25.635.321, PMID: 17736950

[ref43] Jansen N. Kant I. van Amelsvoort L. Nijhuis F. van den Brandt P. (2003). Need for recovery from work: evaluating short-term effects of working hours, patterns and schedules. Ergonomics 46, 664–680. 10.1080/0014013031000085662, PMID: 12745680

[ref44] Jansen N. W. Kant I. van den Brandt P. A. (2002). Need for recovery in the working population: description and associations with fatigue and psychological distress. Int. J. Behav. Med. 9, 322–340. 10.1207/S15327558IJBM0904_03, PMID: 12512472

[ref45] Kahneman D. (1973). Attention and Effort. Englewood Cliffs, NJ: Prentice-Hall.

[ref46] Kanfer R. Ackerman P. L. Murtha T. C. Dugdale B. Nelson L. (1994). Goal setting, conditions of practice, and task performance: a resource allocation perspective. J. Appl. Psychol. 79, 826–835. 10.1037/0021-9010.79.6.826

[ref47] Kool W. Botvinick M. (2014). A labor/leisure tradeoff in cognitive control. Motiv. Sci. 1, 3–18. 10.1037/2333-8113.1.S.3 PMC3739999 23230991

[ref48] Kool W. Botvinick M. (2018). Mental labour. Nat. Hum. Behav. 2, 899–908. 10.1038/s41562-018-0401-9, PMID: 30988433

[ref49] Kool W. McGuire J. T. Rosen Z. B. Botvinick M. M. (2010). Decision making and the avoidance of cognitive demand. J. Exp. Psychol. Gen. 139, 665–682. 10.1037/a0020198, PMID: 20853993 PMC2970648

[ref50] Kruglanski A. W. Bélanger J. J. Chen X. Köpetz C. Pierro A. Mannetti L. (2012). The energetics of motivated cognition: a force-field analysis. Psychol. Rev. 119, 1–20. 10.1037/a0025488, PMID: 21967165

[ref51] Kruglanski A. W. Shah J. Y. Fishbach A. Friedman R. S. Chun W. Y. Sleeth-Keppler D. (2002). “A theory of goal systems,” in Advances in Experimental Social Psychology. ed. Zanna M. P. (San Diego: Academic Press), 331–378.

[ref52] Kurniawan I. T. Guitart-Masip M. Dolan R. J. (2011). Dopamine and effort-based decision making. Front. Neurosci. 5:81. 10.3389/fnins.2011.00081, PMID: 21734862 PMC3122071

[ref53] Kurzban R. (2016). The sense of effort. Curr. Opin. Psychol. 7, 67–70. 10.1016/j.copsyc.2015.08.003

[ref54] Kurzban R. Duckworth A. Kable J. W. Myers J. (2013). An opportunity cost model of subjective effort and task performance. Behav. Brain Sci. 36, 661–679. 10.1017/S0140525X12003196, PMID: 24304775 PMC3856320

[ref55] Laran J. Buechel E. (2017). Mental resources increase preference for dissimilar experiences. J. Assoc. Consum. Res. 2, 123–135. 10.1086/688859

[ref56] Laran J. Janiszewski C. (2011). Work or fun? How task construal and completion influence regulatory behavior. J. Consum. Res. 37, 967–983. 10.1086/656576

[ref57] Latham G. P. Ganegoda D. B. Locke E. A. (2011). “A state theory, but related to traits,” in The Wiley-Blackwell Handbook of Individual Differences. eds. Chamorro-Premuzic T. von Stumm S. Furnham A. (Chichester, UK: Wiley Blackwell), 579–587.

[ref58] Lieberman H. R. (2006). Mental energy: assessing the cognitive dimension. Nutr. Rev. 64, S10–S13. 10.1111/j.1753-4887.2006.tb00252.x 16910216

[ref59] Lieberman H. R. (2007). Cognitive methods for assessing mental energy. Nutr. Neurosci. 10, 229–242. 10.1080/10284150701722273, PMID: 18284031

[ref60] Loy B. D. Cameron M. H. O’Connor P. J. (2018). Perceived fatigue and energy are independent unipolar states: Supporting evidence. Med. Hypotheses 113, 46–51. 10.1016/j.mehy.2018.02.014, PMID: 29523293 PMC5846196

[ref61] Manelis A. Reder L. M. (2015). He who is well prepared has half won the battle: an fMRI study of task preparation. Cereb. Cortex 25, 726–735. 10.1093/cercor/bht262, PMID: 24092642 PMC4318533

[ref62] Maridakis V. Herring M. P. O’Connor P. J. (2009). Sensitivity to change in cognitive performance and mood measures of energy and fatigue in response to differing doses of caffeine or breakfast. Int. J. Neurosci. 119, 975–994. 10.1080/00207450802333995, PMID: 19466633

[ref63] Masicampo E. J. Martin S. R. Anderson R. A. (2014). Understanding and overcoming self-control depletion. Soc. Personal. Psychol. Compass 8, 638–649. 10.1111/spc3.12139

[ref64] McGuire J. T. Botvinick M. M. (2010). Prefrontal cortex, cognitive control, and the registration of decision costs. Proc. Natl. Acad. Sci. U. S. A. 107, 7922–7926. 10.1073/pnas.0910662107, PMID: 20385798 PMC2867898

[ref66] Navon D. Gopher D. (1979). On the economy of the human-processing system. Psychol. Rev. 86, 214–255. 10.1037/0033-295X.86.3.214

[ref67] O’Connor P. J. (2004). Evaluation of four highly cited energy and fatigue mood measures. J. Psychosom. Res. 57, 435–441. 10.1016/j.jpsychores.2003.12.006, PMID: 15581646

[ref68] O’Connor P. J. (2006a). Mental energy: Developing a model for examining nutrition-related claims. Nutr. Rev. 64, S2–S6. 10.1301/nr.2006.jul.s2-s6, PMID: 16910214

[ref69] O’Connor P. J. (2006b). Mental energy: Assessing the mood dimension. Nutr. Rev. 64, S7–S9. 10.1111/j.1753-4887.2006.tb00256.x, PMID: 16910215

[ref70] Robinson M. D. Schmeichel B. J. Inzlicht M. (2010). A cognitive control perspective of self-control strength and its depletion. Soc. Personal. Psychol. Compass 4, 189–200. 10.1111/j.1751-9004.2009.00244.x

[ref71] Sayalı C. Badre D. (2019). Neural systems of cognitive demand avoidance. Neuropsychologia 123, 41–54. 10.1016/j.neuropsychologia.2018.06.016, PMID: 29944865 PMC6317343

[ref72] Schmeichel B. J. Vohs K. D. Baumeister R. F. (2003). Intellectual performance and ego depletion: role of the self in logical reasoning and other information processing. J. Pers. Soc. Psychol. 85, 33–46. 10.1037/0022-3514.85.1.33, PMID: 12872883

[ref73] See J. E. Howe S. R. Warm J. S. Dember W. N. (1995). Meta-analysis of the sensitivity decrement in vigilance. Psychol. Bull. 117, 230–249. 10.1037/0033-2909.117.2.230

[ref74] Shenhav A. Botvinick M. M. Cohen J. D. (2013). The expected value of control: an integrative theory of anterior cingulate cortex function. Neuron 79, 217–240. 10.1016/j.neuron.2013.07.007, PMID: 23889930 PMC3767969

[ref75] Shenhav A. Musslick S. Lieder F. Kool W. Griffiths T. L. Cohen J. D.. (2017). Toward a rational and mechanistic account of mental effort. Annu. Rev. Neurosci. 40, 99–124. 10.1146/annurev-neuro-072116-031526, PMID: 28375769

[ref76] Shulmana R. G. Hydera F. Rothmana D. L. (2009). Baseline brain energy supports the state of consciousness. Proc. Natl. Acad. Sci. U. S. A. 106, 11096–11101. 10.1073/pnas.0903941106, PMID: 19549837 PMC2708743

[ref77] van der Linden D. Frese M. Meijman T. F. (2003). Mental fatigue and the control of cognitive processes: effects on perseveration and planning. Acta Psychol. 113, 45–65. 10.1016/S0001-6918(02)00150-6 12679043

[ref78] van Veldhoven M. J. P. M. Broersen S. (2003). Measurement quality and validity of the “need for recovery scale.” Occup. Environ. Med. 60(Suppl. 1), i3–i9. 10.1136/oem.60.suppl_1.i3, PMID: 12782740 PMC1765728

[ref79] Vohs K. D. Baumeister R. F. Ciarocco N. J. (2005). Self-regulation and self-presentation: regulatory resource depletion impairs impression management and effortful self-presentation depletes regulatory resources. J. Pers. Soc. Psychol. 88, 632–657. 10.1037/0022-3514.88.4.632, PMID: 15796665

[ref80] Vohs K. D. Baumeister R. F. Schmeichel B. J. Twenge J. M. Nelson N. M. Tice D. M. (2014). Making choices impairs subsequent self-control: a limited-resource account of decision making, self-regulation, and active initiative. Motiv. Sci. 1, 19–42. 10.1037/2333-8113.1.S.19 18444745

[ref81] Vohs K. D. Heatherton T. F. (2000). Self-regulatory failure: a resource-depletion approach. Psychol. Sci. 11, 249–254. 10.1111/1467-9280.00250, PMID: 11273412

[ref83] Wang J. Novemsky N. Dhar R. Baumeister R. F. (2010). Trade-offs and depletion in choice. J. Mark. Res. 47, 910–919. 10.1509/jmkr.47.5.910

[ref84] Ward-Ritacco C. Poudevigne M. S. O’Connor P. J. (2016). Muscle strengthening exercises during pregnancy are associated with increased energy and reduced fatigue. J. Psychosom. Obstet. Gynecol. 37, 68–72. 10.3109/0167482X.2016.1155552, PMID: 26984583 PMC4978349

[ref85] Wigfield A. Eccles J. S. (2000). Expectancy–value theory of achievement motivation. Contemp. Educ. Psychol. 25, 68–81. 10.1006/ceps.1999.1015, PMID: 10620382

[ref86] Wingfield A. (2016). Evolution of models of working memory and cognitive resources. Ear Hear. 37, 35S–43S. 10.1097/AUD.0000000000000310, PMID: 27355768

[ref87] Wood C. Magnello M. E. (1992). Diurnal changes in perceptions of energy and mood. J. R. Soc. Med. 85, 191–194. PMID: 1433057 10.1177/014107689208500404 PMC1294720

[ref88] Yeo G. Neal A. (2008). Subjective cognitive effort: a model of states, traits, and time. J. Appl. Psychol. 93, 617–631. 10.1037/0021-9010.93.3.617, PMID: 18457490

