# Supplementary material for: Deriving Mental Energy From Task Completion
Source: Front Psychol. 2021 Aug 20;12:717414. doi: 10.3389/fpsyg.2021.717414 (PMC8418126; doi:10.3389/fpsyg.2021.717414)
Supplement: Supplementary file 1 [file Data_Sheet_1.docx]

Supplementary Material

Table of Contents

[Study 1 Stimuli 2](#_Toc62037454)

[Study 1 Replicaton Using a Low Reward Condition 9](#_Toc62037455)

[Study 2 Stimuli 11](#_Toc62037456)

[Study 3 Stimuli 26](#_Toc62037457)

[Study 4 Stimuli 42](#_Toc62037458)

[Study 4 Results: Means of T1 through T6 51](#_Toc62037459)

# Study 1 Stimuli

2 (reward value: low vs. high), between-subjects

**[Low Reward Condition]**


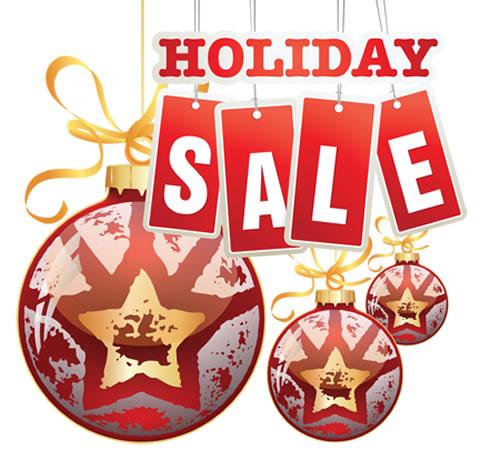


**Holiday Sales Online Now -- Find out the best deal!**

On the next few pages, we will show you several items. Your task is to find the **best deal online** **(the lowest price)** for each item. You will be asked to paste the link of the deal to a textbox and write down the price you find for each item.

The deals you find should not require membership, use of specific credit cards/gift cards, purchase of other items (e.g., BOGO 50% off, $50 off $300 purchase if the price of the item is lower than $300). Deals may include price markdowns, extra savings/discounts, cashback, rebates, etc.

**Please make sure that the deal you find applies to EXACTLY THE SAME ITEM we show you. We will check your answers after you submitted the HIT.**

Ready? Click >> to start!

**[High Reward Condition]**


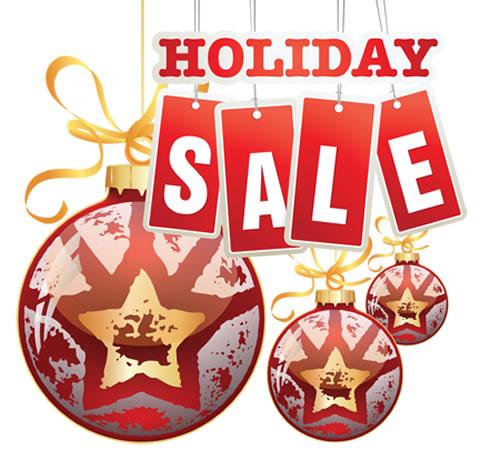


**Holiday Sales Online Now -- Find out the best deal!**

On the next few pages, we will show you several items. Your task is to find the **best deal online** **(the lowest price)** for each item. You will be asked to paste the link of the deal to a textbox and write down the price you find for each item.

The deals you find should not require membership, use of specific credit cards/gift cards, purchase of other items (e.g., BOGO 50% off, $50 off $300 purchase if the price of the item is lower than $300). Deals may include price markdowns, extra savings/discounts, cashback, rebates, etc.

**Please make sure that the deal you find applies to EXACTLY THE SAME ITEM we show you. We will check your answers after you submitted the HIT.**

At the end of the survey, we will show you the best deals we found. For each item, if the deal you found matches ours, or the price you indicated is lower than our price, you will get a **$0.1 bonus/per item.**

Ready? Click >> to start!

Page Break


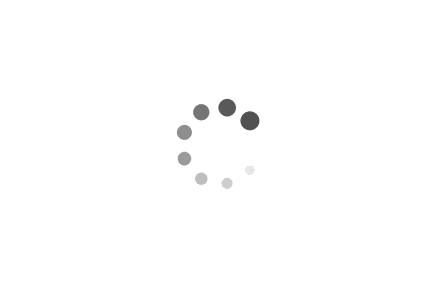


Loading the first item

Page Break

[T1]

Before you search for the first deal, we would like to know how much mental energy you have at this moment.

***People's mental energy fluctuates on a moment-to-moment basis. We will ask you to indicate how much energy you have at various time in this study.*

On the following scale, please indicate how much energy you feel you have AT THIS MOMENT. (1 = Less energy than usual, 7 = More energy than usual)

Page Break

Thank you! Now please click >> to see the first item you will search for.

Page Break


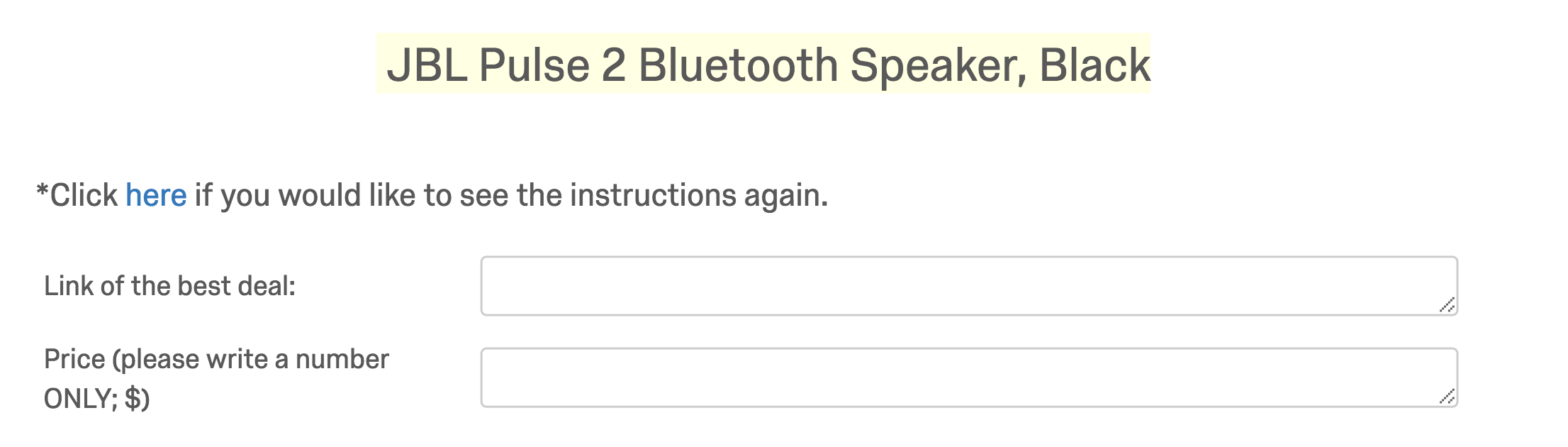


Page Break


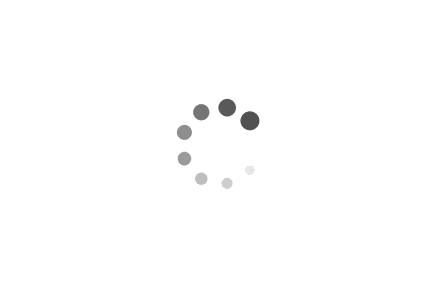


Loading next item

Page Break

[T2]

Before you search for the next deal, we would like to know how much mental energy you have at this moment. People's mental energy fluctuates on a moment-to-moment basis. On the following scale, please indicate how much energy you feel you have at this moment. (1 = Less energy than usual, 7 = More energy than usual)

Page Break

Thank you! Now please click >> to continue with the task.

Page Break


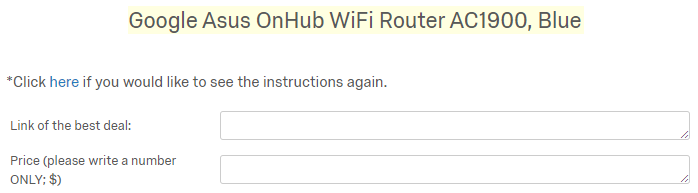


Page Break


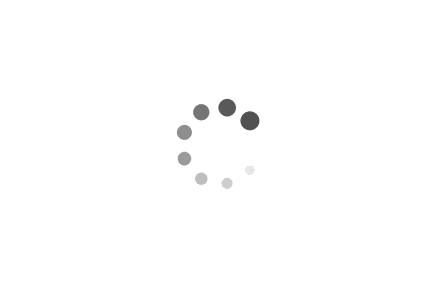


Loading next item

Page Break

[T3]

Before you search for the next deal, we would like to know how much mental energy you have at this moment. People's mental energy fluctuates on a moment-to-moment basis. On the following scale, please indicate how much energy you feel you have at this moment. (1 = Less energy than usual, 7 = More energy than usual)

Page Break

Thank you! Now please click >> to continue with the task.

Page Break


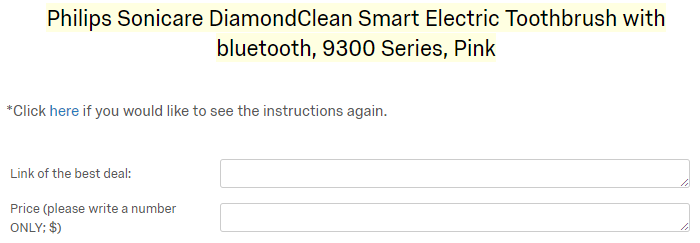


Page Break


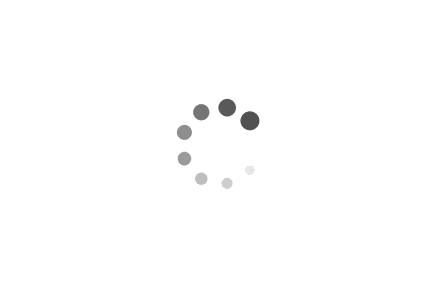


Loading next item

Page Break

[T4]

Before you search for the next deal, we would like to know how much mental energy you have at this moment. People's mental energy fluctuates on a moment-to-moment basis. On the following scale, please indicate how much energy you feel you have at this moment. (1 = Less energy than usual, 7 = More energy than usual)

Page Break

Thank you! Now please click >> to continue with the task.

Page Break


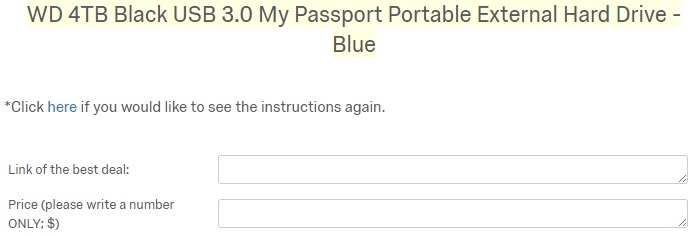


Page Break


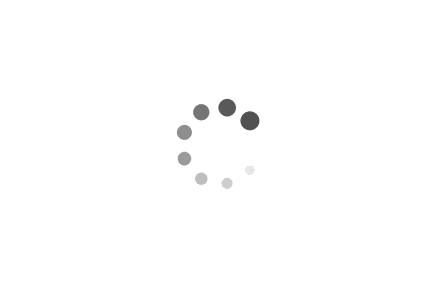


Loading next item

Page Break

[T5]

Before you search for the next deal, we would like to know how much mental energy you have at this moment. People's mental energy fluctuates on a moment-to-moment basis. On the following scale, please indicate how much energy you feel you have at this moment. (1 = Less energy than usual, 7 = More energy than usual)

Page Break

Thank you! Now please click >> to continue with the task.

Page Break


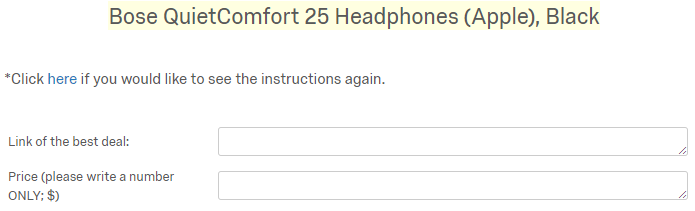


Page Break


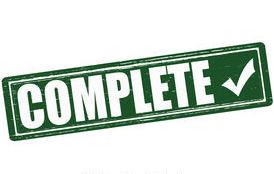


Congratulations! You have completed the tasks.

[T6]

On the following scale, please indicate how much energy you feel you have at this moment. (1 = Less energy than usual, 7 = More energy than usual)

Page Break

[Felt achievement measure]

To what extent do you think completing the find-the-best-deal task felt like an achievement? (1 = not at all, 7 = very much)

[Competence measure]

How effective did you feel at the find-the-best-deal task? (1 = not at all, 7 = very much)

# Study 1 Replication Using a Low Reward Condition

## Method

A total of 118 Mechanical Turk participants completed the study in exchange for $1.20 in financial compensation. The experiment used a two cell (reward value: low vs. high), between-subject design. The procedure was identical to that of study 1, except that a) participants in the low reward condition learned that they would receive a $0.01 bonus for each best deal they found, b) we removed holiday-related words and pictures in the instructions as in study 2 (i.e., the instructions were identical to those in study 2), and c) the specifics of some products were changed (e.g., color, model type) due to product availability or deal availability.

## Results

**Mental energy***.* As in Study 1, a repeated measures ANOVA with reward value (low vs. high) as a between-subjects factor and time (T5 vs. T6) as a within-subjects factor showed a significant interaction between time (T5 vs. T6) and reward value (*F*(1, 116) = 4.38, *p* = .039, ω_p_^2^ = .028). Follow-up pairwise comparisons revealed that participants in the high-reward condition exhibited higher mental energy replenishment (*M*_T5_ = 4.33, SD = 1.42; *M*_T6_ = 4.62, SD = 1.41; *F*(1, 116) = 7.71, *p* = .006, ω_p_^2^ = .054). However, participants in the low-reward condition exhibited no mental energy replenishment (*M*_T5_ = 4.32, SD = 1.66; *M*_T6_ = 4.30, SD = 1.74; *F*(1, 116) = .03, *p* = .873).

**Felt Achievement and Competence**. The effect of reward value on felt achievement and competence was directional, but not significant (felt achievement: *M*_low_ = 4.15, *SD*_low_ = 1.83; *M*_high_ = 4.67, *SD*_high_ = 1.61; *F*(1, 116) = 2.71, *p* = .102; competence: *M*_low_ = 4.67, *SD*_low_ = 1.53; *M*_high_ = 4.84, *SD*_high_ = 1.36; *F*(1, 116) = .45, *p* = .505). A follow-up test showed the interaction between time (T6 – T5) and reward on mental energy remained significant after controlling for felt achievement and competence (*F*(1, 114) = 4.51, *p* = .036). Thus, consistent with the conclusions of study 1, felt achievement and competence could not account for the effect of reward on mental energy replenishment.

# Study 2 Stimuli

2 (reward value: low vs. high) * 2(completion: yes vs. no), between-subjects

**[Low Reward Condition]**


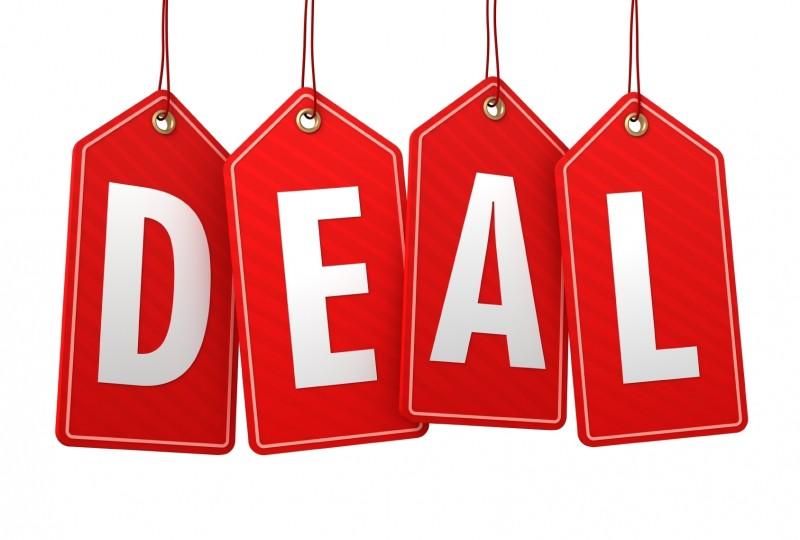
​

**Find out the best deal!**

On the next few pages, we will show you several items. Your task is to find the **best deal online** **(the lowest price)** for each item. You will be asked to paste the link of the deal to a textbox and write down the price you find for each item.

The deals you find should not require membership, use of specific credit cards/gift cards, purchase of other items (e.g., BOGO 50% off, $50 off $300 purchase if the price of the item is lower than $300). Deals may include price markdowns, extra savings/discounts, cashback, rebates, etc.

**Please make sure that the deal you find applies to EXACTLY THE SAME ITEM we show you. We will check your answers after you submitted the HIT.**

Ready? Click >> to start!

**[High Reward Condition]**


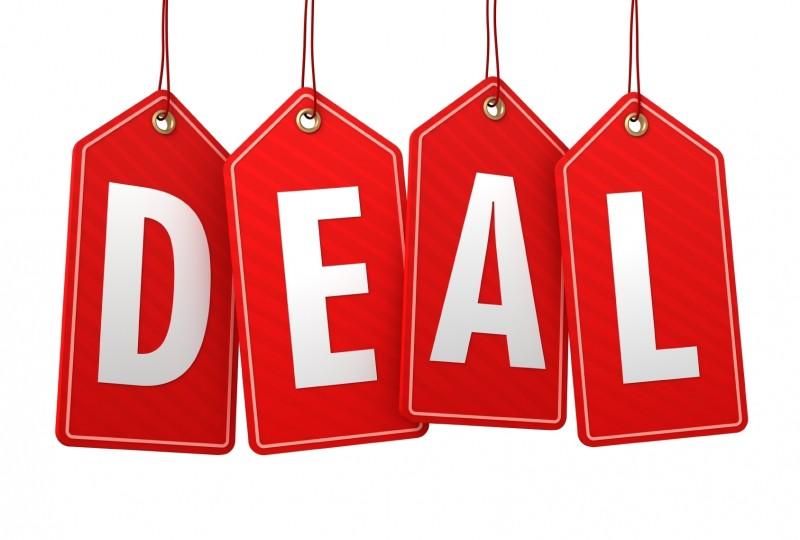
​

**Find out the best deal!**

On the next few pages, we will show you several items. Your task is to find the **best deal online** **(the lowest price)** for each item. You will be asked to paste the link of the deal to a textbox and write down the price you find for each item.

The deals you find should not require membership, use of specific credit cards/gift cards, purchase of other items (e.g., BOGO 50% off, $50 off $300 purchase if the price of the item is lower than $300). Deals may include price markdowns, extra savings/discounts, cashback, rebates, etc.

**Please make sure that the deal you find applies to EXACTLY THE SAME ITEM we show you. We will check your answers after you submitted the HIT.**

At the end of the survey, we will show you the best deals we found. For each item, if the deal you found matches ours, or the price you indicated is lower than our price, you will get a **$0.1 bonus/per item.**

Ready? Click >> to start!

Page Break


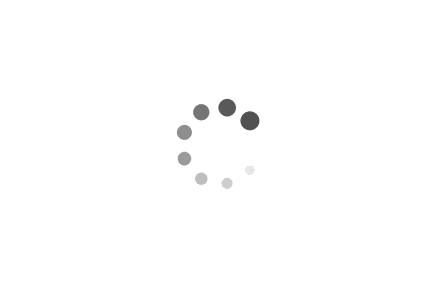


Loading the first item

Page Break


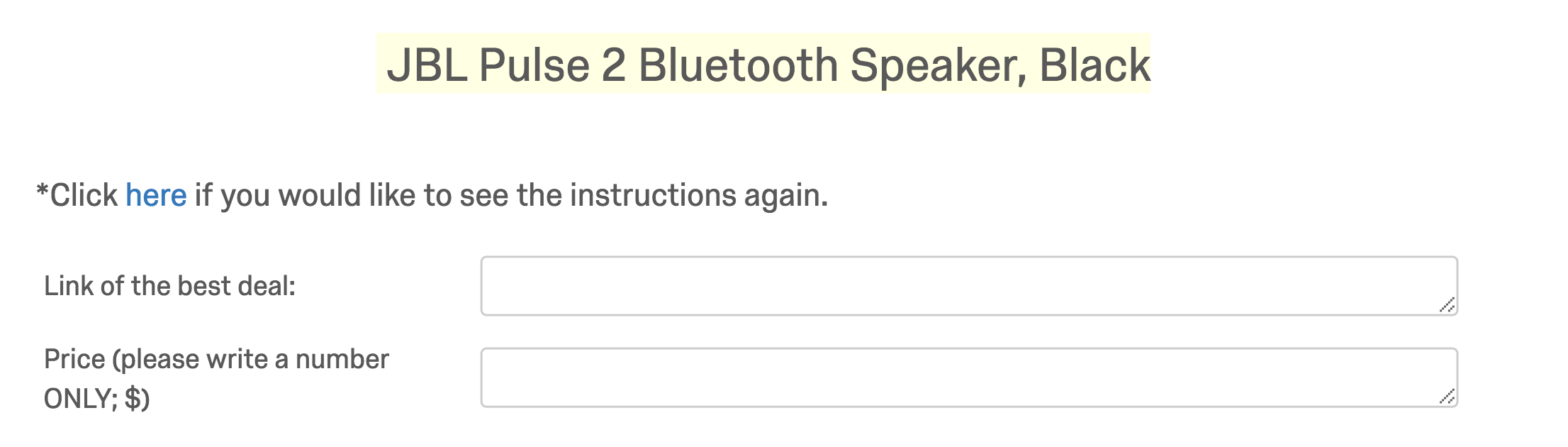


Page Break


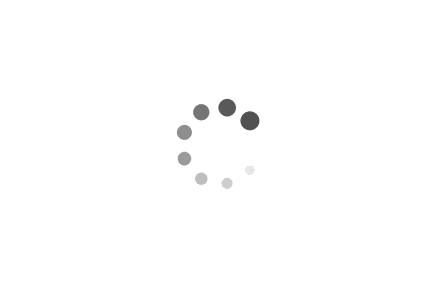


Loading next item

Page Break


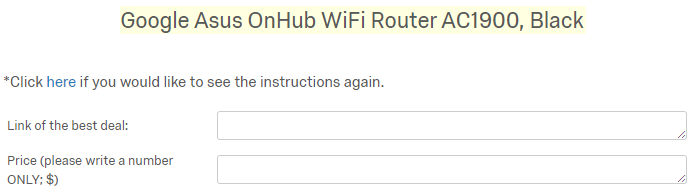


Page Break


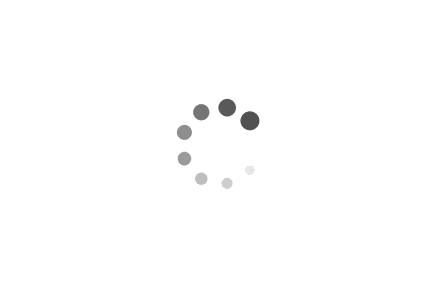


Loading next item

Page Break


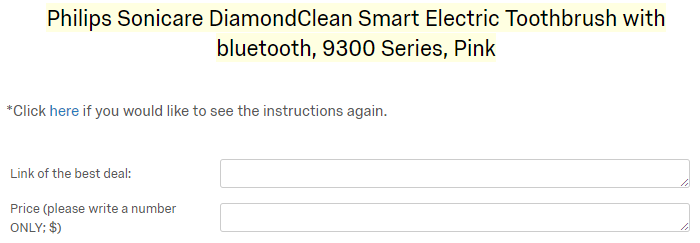


Page Break


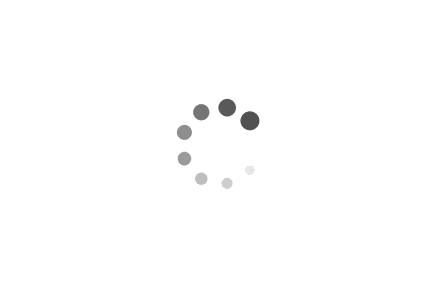


Loading next item

Page Break


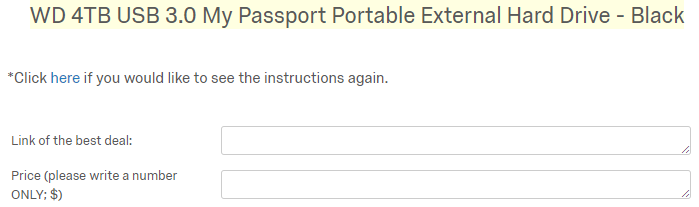


Page Break


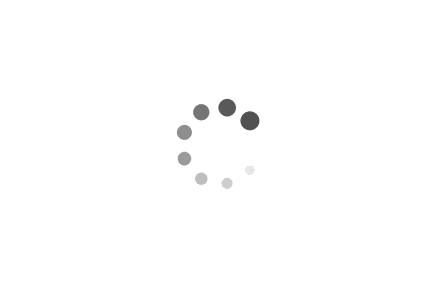


Loading next item

Page Break


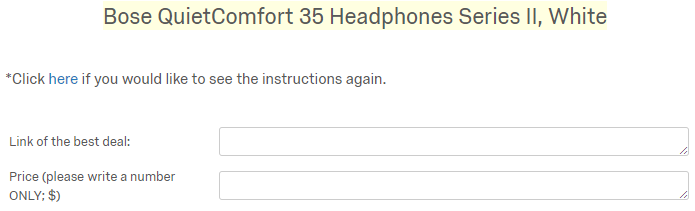


Page Break

**[Completion condition]**


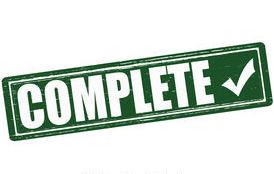


Congratulations! You have completed this task.

Page Break

**Now we would like you to complete another task.**

Page Break

**[No completion condition]**


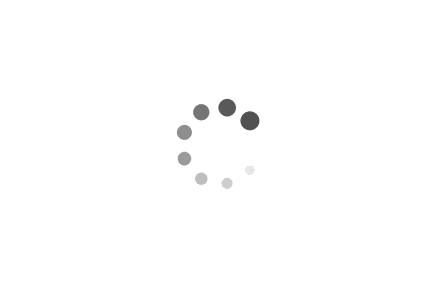


Loading next item

Page Break

**Now we would like you to switch to another task.**

Page Break

Book Evaluation Task

On the next few pages, we would like you to evaluate some newly released books and tell us whether you will consider adding them to your reading list. On each page, we will show you the book title, author, and a synopsis. After you evaluate some books, you can choose to quit the task. You can quit the task whenever you like.

Page Break

Please read the book information below.

**
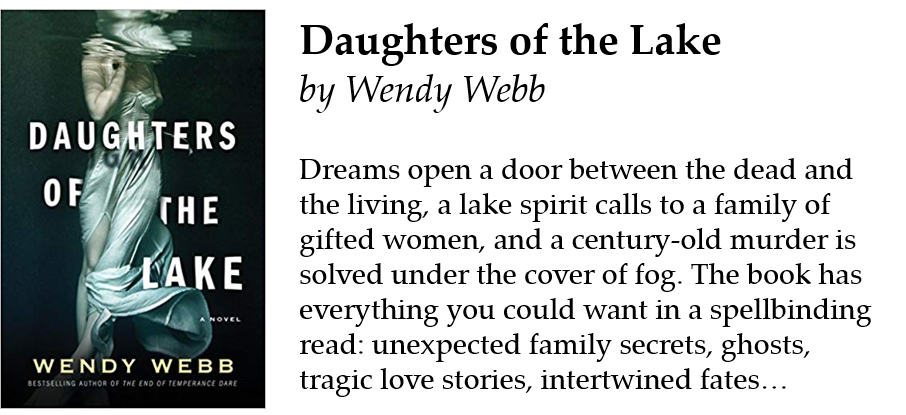
**

Would you consider adding this book to your reading list? (“Yes” or “No”)

To what extent are you interested in reading this book? (1 = not at all, 7 = Very much)

Would you like to quit the task or continue working on the task (i.e., evaluate more books)? (“Quit” or “Continue working on this task”)

*[If “continue working on this task” is chosen]*

Please read the book information below.

**
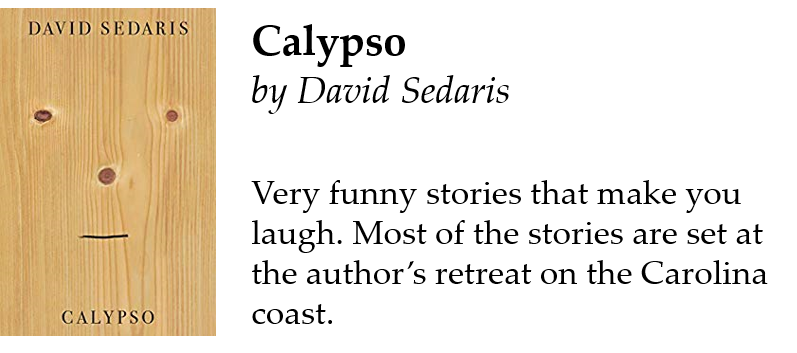
**

Would you consider adding this book to your reading list? (“Yes” or “No”)

To what extent are you interested in reading this book? (1 = not at all, 7 = Very much)

Would you like to quit the task or continue working on the task (i.e., evaluate more books)? (“Quit” or “Continue working on this task”)

*[If “continue working on this task” is chosen]*

Please read the book information below.

**
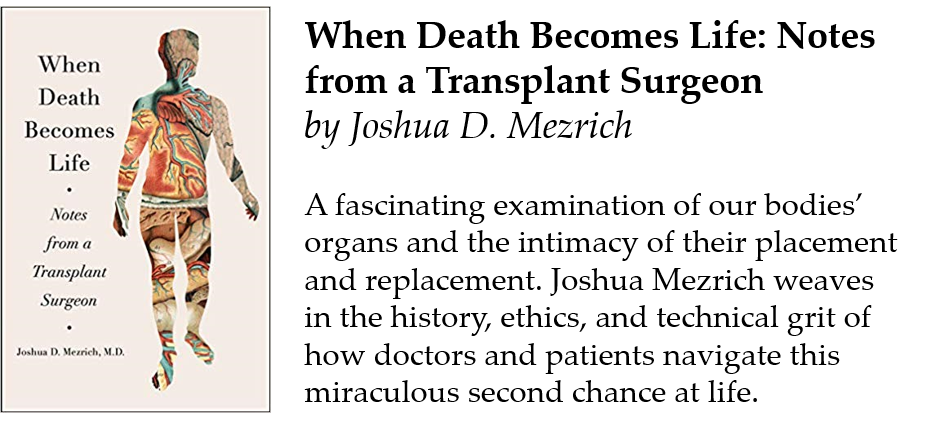
**

Would you consider adding this book to your reading list? (“Yes” or “No”)

To what extent are you interested in reading this book? (1 = not at all, 7 = Very much)

Would you like to quit the task or continue working on the task (i.e., evaluate more books)? (“Quit” or “Continue working on this task”)

Page Break

*[If “continue working on this task” is chosen]*

Please read the book information below.

**
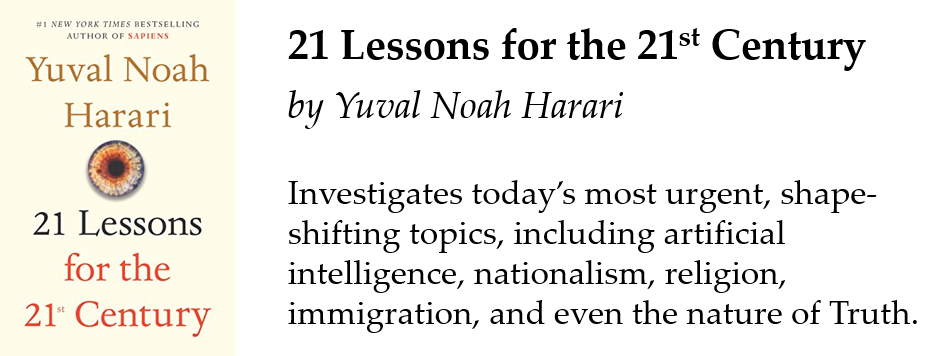
**

Would you consider adding this book to your reading list? (“Yes” or “No”)

To what extent are you interested in reading this book? (1 = not at all, 7 = Very much)

Would you like to quit the task or continue working on the task (i.e., evaluate more books)? (“Quit” or “Continue working on this task”)

Page Break

*[If “continue working on this task” is chosen]*

Please read the book information below.

**
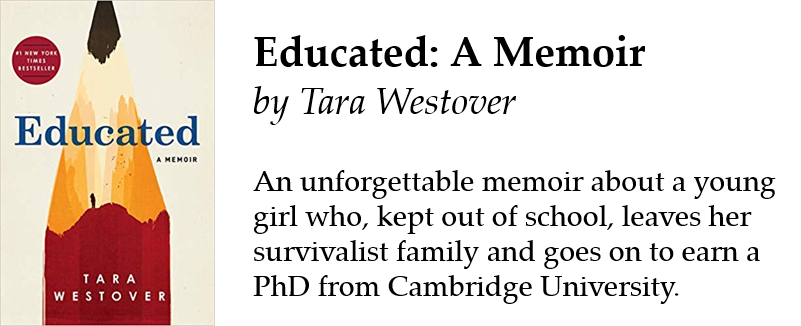
**

Would you consider adding this book to your reading list? (“Yes” or “No”)

To what extent are you interested in reading this book? (1 = not at all, 7 = Very much)

Would you like to quit the task or continue working on the task (i.e., evaluate more books)? (“Quit” or “Continue working on this task”)

Page Break

*[If “continue working on this task” is chosen]*

Please read the book information below.

**
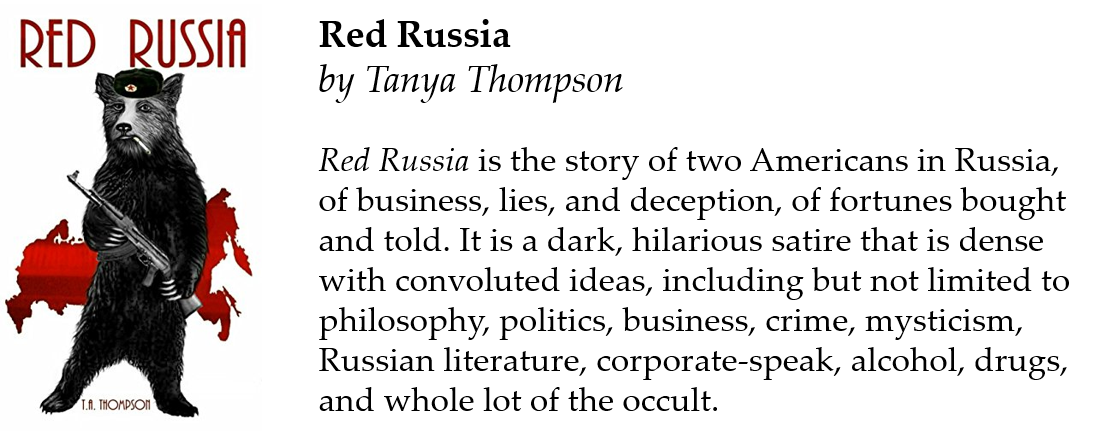
**

Would you consider adding this book to your reading list? (“Yes” or “No”)

To what extent are you interested in reading this book? (1 = not at all, 7 = Very much)

Would you like to quit the task or continue working on the task (i.e., evaluate more books)? (“Quit” or “Continue working on this task”)

Page Break

*[If “continue working on this task” is chosen]*

Please read the book information below.

**
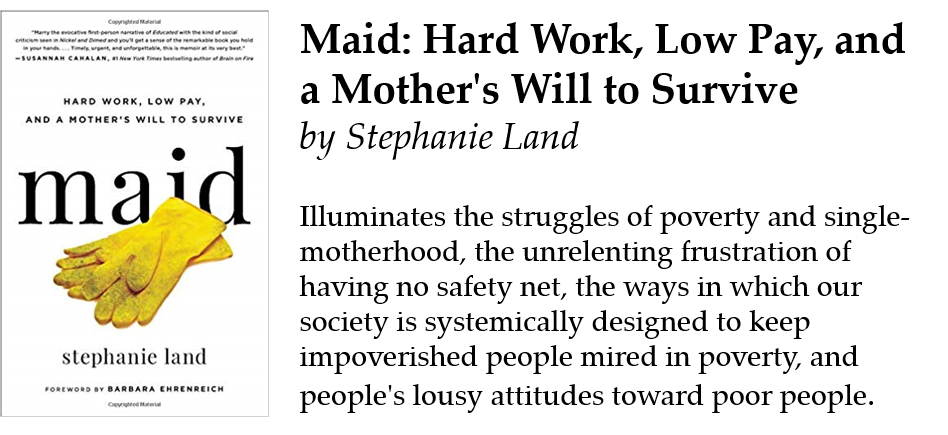
**

Would you consider adding this book to your reading list? (“Yes” or “No”)

To what extent are you interested in reading this book? (1 = not at all, 7 = Very much)

Would you like to quit the task or continue working on the task (i.e., evaluate more books)? (“Quit” or “Continue working on this task”)

Page Break

*[If “continue working on this task” is chosen]*

Please read the book information below.

**
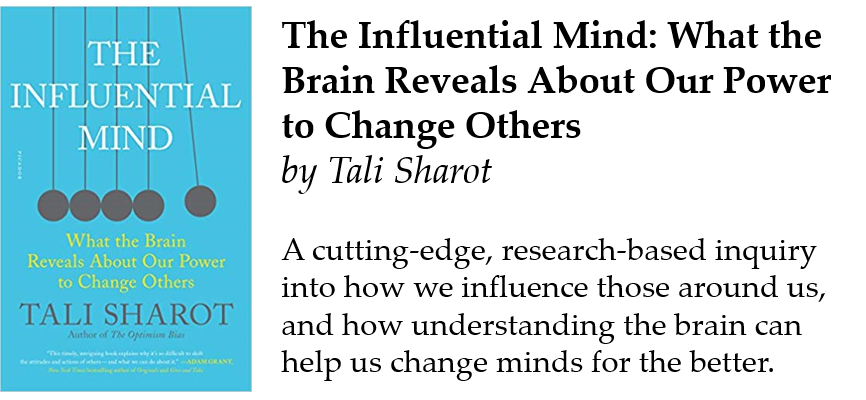
**

Would you consider adding this book to your reading list? (“Yes” or “No”)

To what extent are you interested in reading this book? (1 = not at all, 7 = Very much)

Would you like to quit the task or continue working on the task (i.e., evaluate more books)? (“Quit” or “Continue working on this task”)

Page Break

*[If “continue working on this task” is chosen]*

Please read the book information below.

**
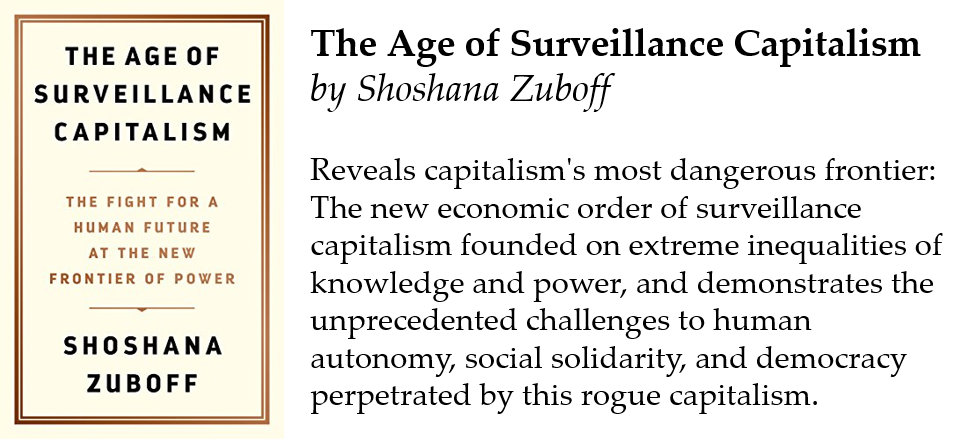
**

Would you consider adding this book to your reading list? (“Yes” or “No”)

To what extent are you interested in reading this book? (1 = not at all, 7 = Very much)

Would you like to quit the task or continue working on the task (i.e., evaluate more books)? (“Quit” or “Continue working on this task”)

Page Break

*[If “continue working on this task” is chosen]*

Please read the book information below.

**
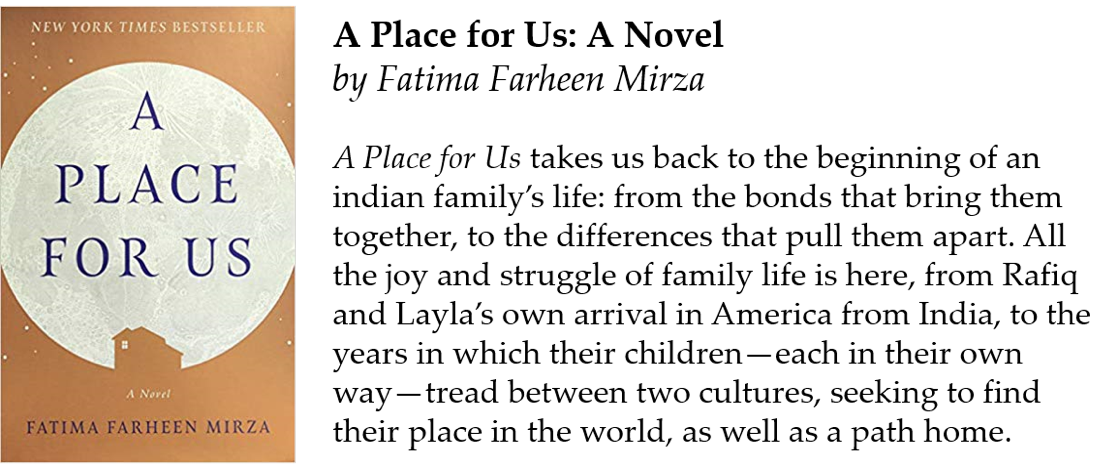
**

Would you consider adding this book to your reading list? (“Yes” or “No”)

To what extent are you interested in reading this book? (1 = not at all, 7 = Very much)

Would you like to quit the task or continue working on the task (i.e., evaluate more books)? (“Quit” or “Continue working on this task”)

Page Break

*[If “continue working on this task” is chosen]*

Please read the book information below.

**
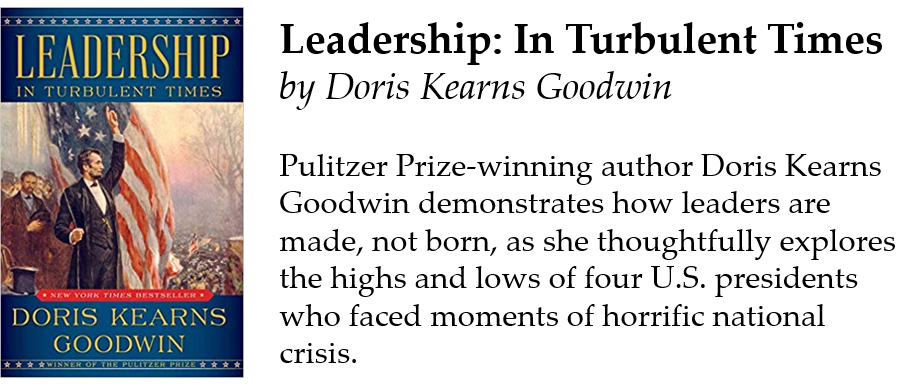
**

Would you consider adding this book to your reading list? (“Yes” or “No”)

To what extent are you interested in reading this book? (1 = not at all, 7 = Very much)

Would you like to quit the task or continue working on the task (i.e., evaluate more books)? (“Quit” or “Continue working on this task”)

Page Break

*[If “continue working on this task” is chosen]*

Please read the book information below.

**
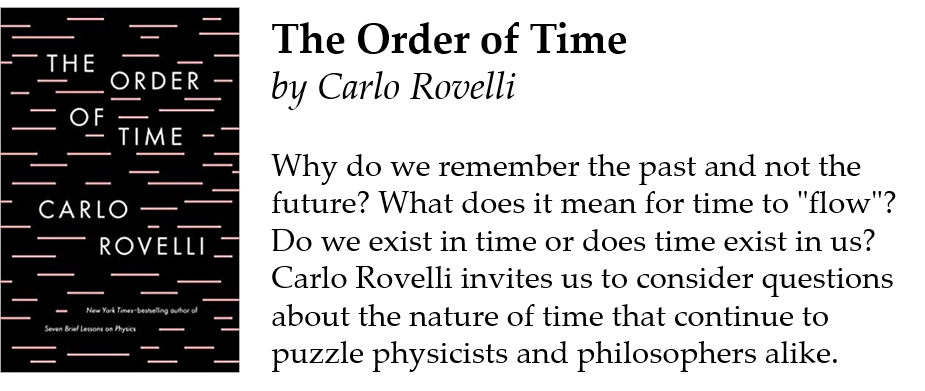
**

Would you consider adding this book to your reading list? (“Yes” or “No”)

To what extent are you interested in reading this book? (1 = not at all, 7 = Very much)

Would you like to quit the task or continue working on the task (i.e., evaluate more books)? (“Quit” or “Continue working on this task”)

Page Break

*[If “continue working on this task” is chosen]*

Please read the book information below.

**
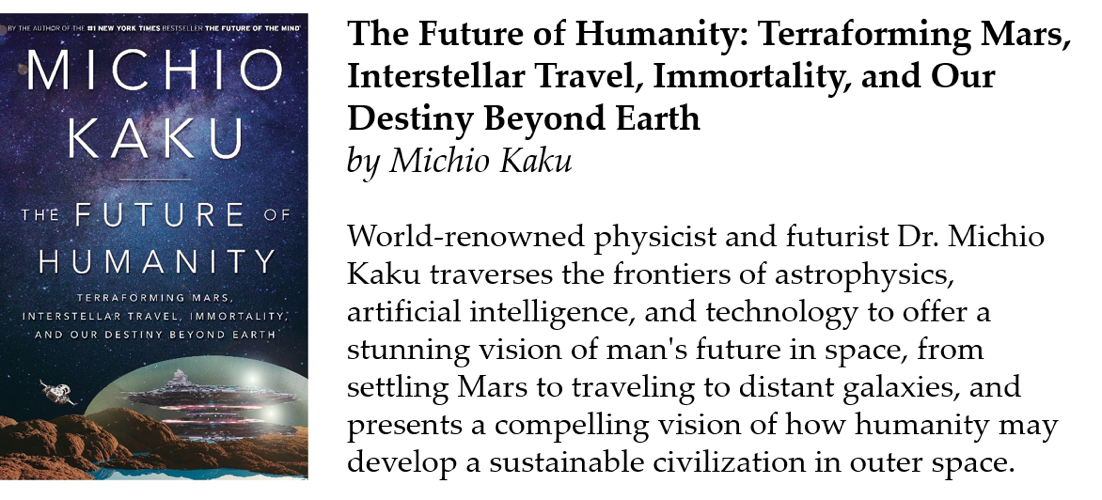
**

Would you consider adding this book to your reading list? (“Yes” or “No”)

To what extent are you interested in reading this book? (1 = not at all, 7 = Very much)

Would you like to quit the task or continue working on the task (i.e., evaluate more books)? (“Quit” or “Continue working on this task”)

Page Break

*[If “continue working on this task” is chosen]*

Please read the book information below.

**
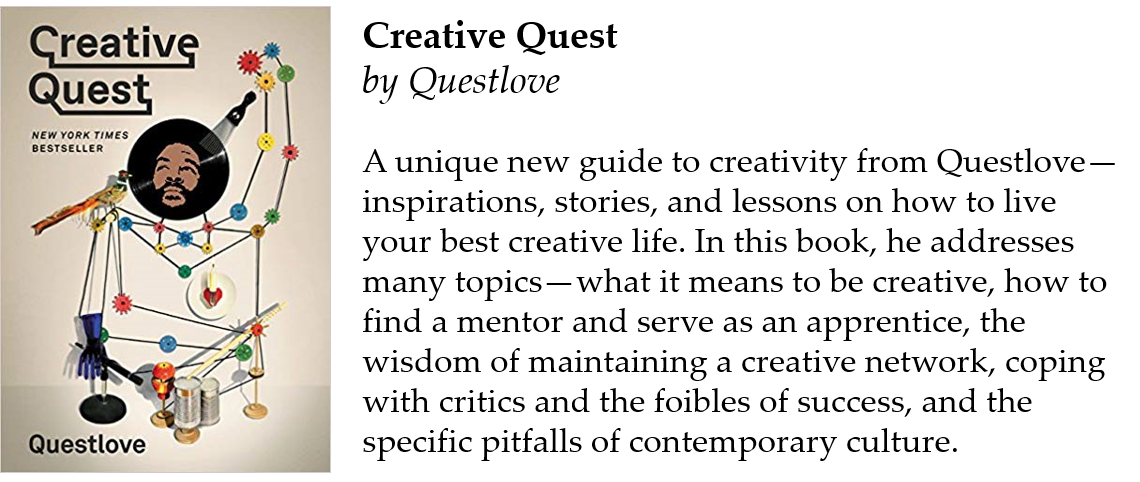
**

Would you consider adding this book to your reading list? (“Yes” or “No”)

To what extent are you interested in reading this book? (1 = not at all, 7 = Very much)

Would you like to quit the task or continue working on the task (i.e., evaluate more books)? (“Quit” or “Continue working on this task”)

Page Break

*[If “continue working on this task” is chosen]*

Please read the book information below.

**
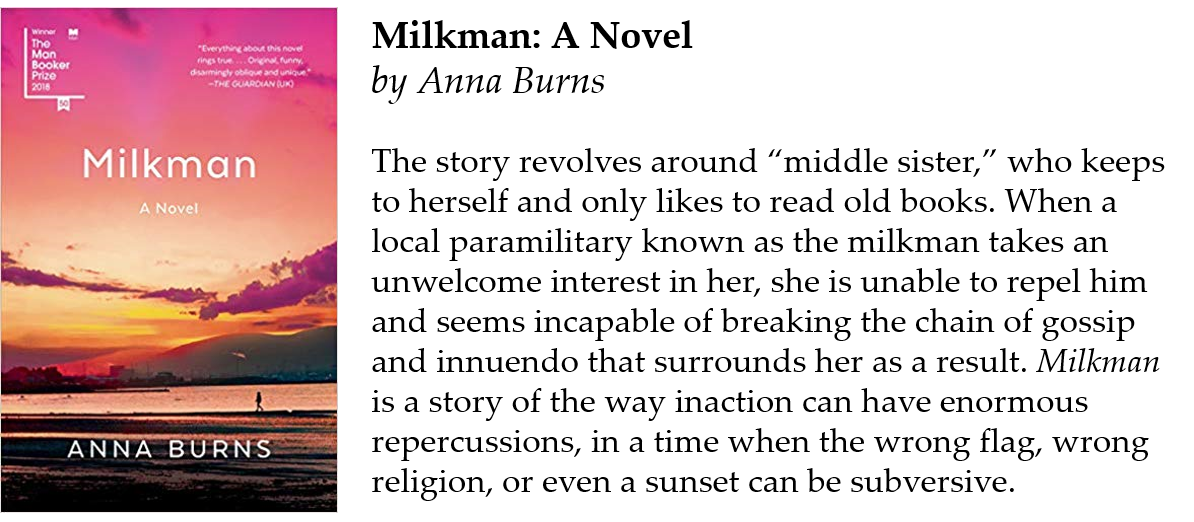
**

Would you consider adding this book to your reading list? (“Yes” or “No”)

To what extent are you interested in reading this book? (1 = not at all, 7 = Very much)

Would you like to quit the task or continue working on the task (i.e., evaluate more books)? (“Quit” or “Continue working on this task”)

Page Break

*[If “continue working on this task” is chosen]*

Please read the book information below.

**
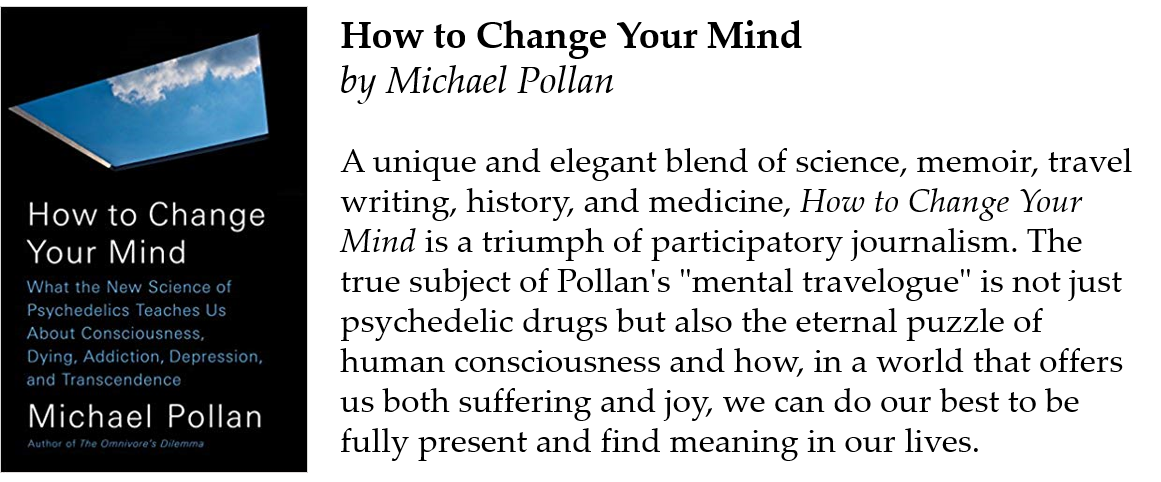
**

Would you consider adding this book to your reading list? (“Yes” or “No”)

To what extent are you interested in reading this book? (1 = not at all, 7 = Very much)

Would you like to quit the task or continue working on the task (i.e., evaluate more books)? (“Quit” or “Continue working on this task”)

Page Break

*[If “continue working on this task” is chosen]*

Please read the book information below.

**
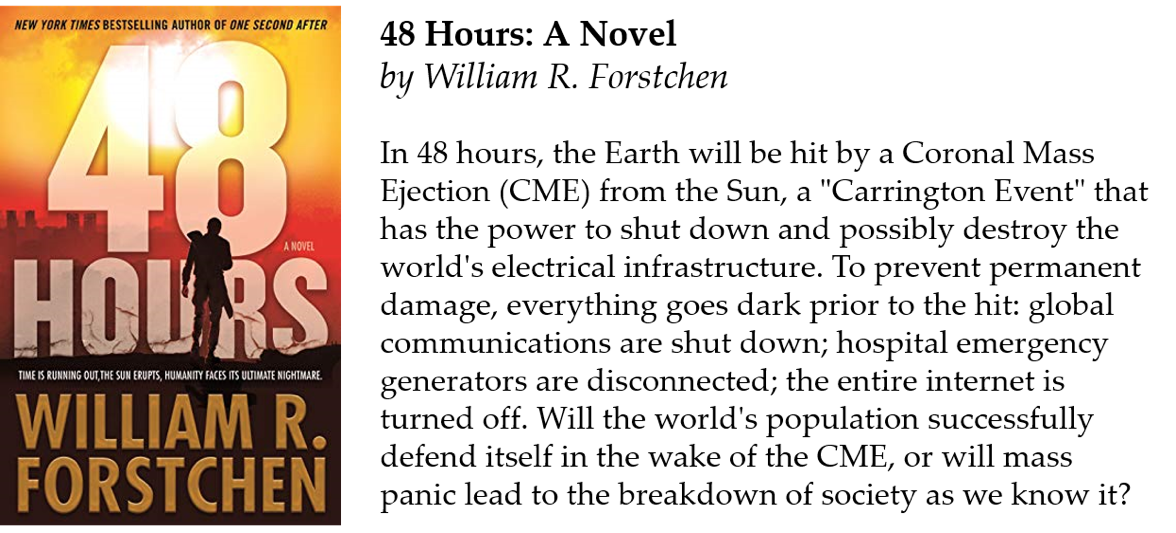
**

Would you consider adding this book to your reading list? (“Yes” or “No”)

To what extent are you interested in reading this book? (1 = not at all, 7 = Very much)

Would you like to quit the task or continue working on the task (i.e., evaluate more books)? (“Quit” or “Continue working on this task”)

Page Break

*[If “continue working on this task” is chosen]*

Please read the book information below.

**
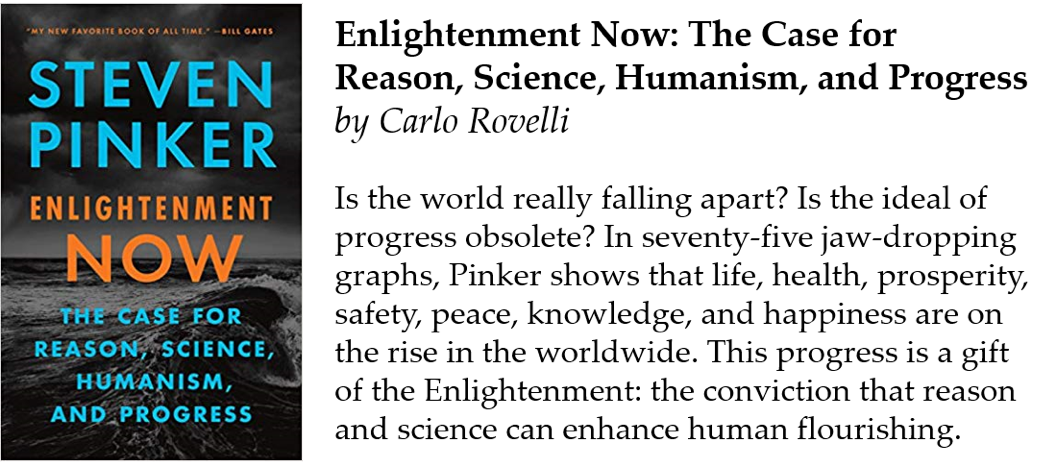
**

Would you consider adding this book to your reading list? (“Yes” or “No”)

To what extent are you interested in reading this book? (1 = not at all, 7 = Very much)

Would you like to quit the task or continue working on the task (i.e., evaluate more books)? (“Quit” or “Continue working on this task”)

Page Break

*[If “continue working on this task” is chosen]*

Please read the book information below.

**
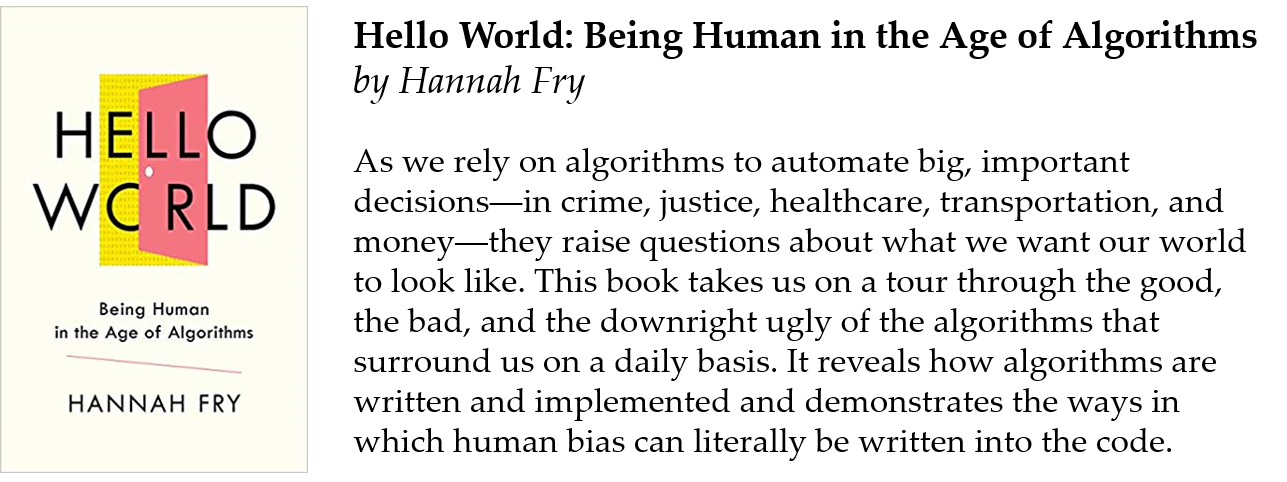
**

Would you consider adding this book to your reading list? (“Yes” or “No”)

To what extent are you interested in reading this book? (1 = not at all, 7 = Very much)

Would you like to quit the task or continue working on the task (i.e., evaluate more books)? (“Quit” or “Continue working on this task”)

# Study 3 Stimuli

2 (reward value: low vs. high) by 2 (expected effort: low vs. high), between-subjects

**[Low reward condition]**


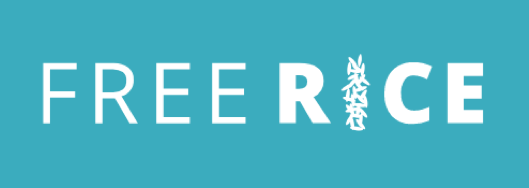


ABOUT

This is a simple task where you will be asked to identify the correct synonym for the displayed word from four alternatives. This task is programmed by Freerice -- a 100% non-profit website that is owned by and supports the United Nations World Food Programme.

The task aims to help end world hunger. For each question you get right, sponsors of Freerice will donate **1 grain of rice** to the United Nations World Food Program to help reach Zero Hunger.


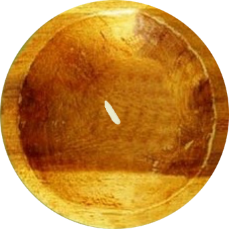


This food is distributed worldwide to those most in need. You can make a real-life contribution to world poverty and society at large.


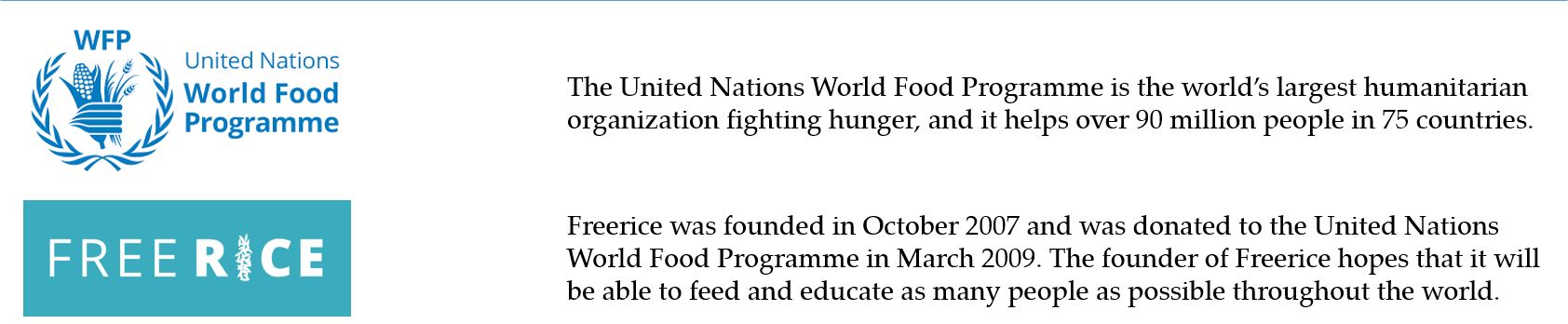


**[High reward condition]**


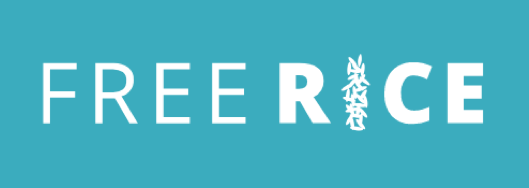


ABOUT

This is a simple task where you will be asked to identify the correct synonym for the displayed word from four alternatives. This task is programmed by Freerice -- a 100% non-profit website that is owned by and supports the United Nations World Food Programme.

The task aims to help end world hunger. For each question you get right, sponsors of Freerice will donate **50 grains of rice** to the United Nations World Food Program to help reach Zero Hunger.


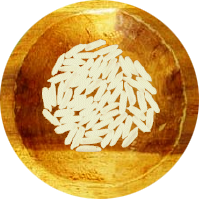


This food is distributed worldwide to those most in need. You can make a real-life contribution to world poverty and society at large.


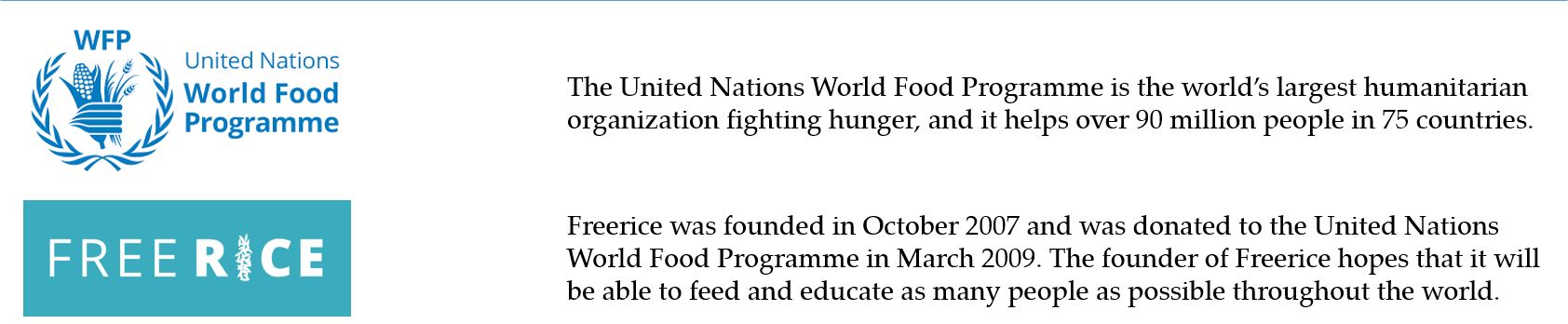


Page Break

**[Low expected effort condition]**


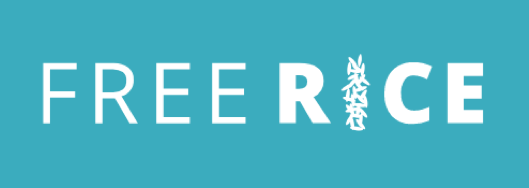


Now you will start working on the task.

How effortful do you think the task will be? (1 = Not at all, 7 = Very effortful)

**[High expected effort condition]**


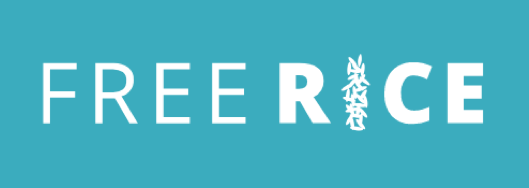


Now you will start working on the task.

**The words you will see in the questions are not those frequently used in everyday life.**

**Thus, you will need to put some effort and think hard.**

How effortful do you think the task will be? (1 = Not at all, 7 = Very effortful)

Page Break


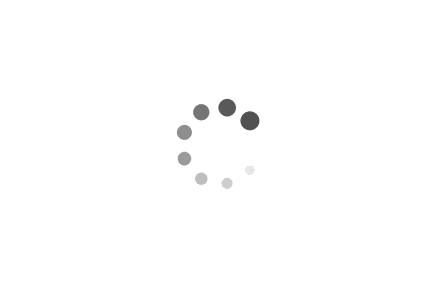


Loading the first question set

Page Break

[T1]

Before you start, we would like to know how much mental energy you have at this moment.

**We need mental energy to get things done. Mental energy is how alert your brain feels at the moment – the degree to which you can remain focused on the very next task.

People’s mental energy fluctuates on a moment-to-moment basis. We will ask you to indicate how much mental energy you have at various time in this study.

On the following scale, please indicate how much mental energy you feel you have AT THIS MOMENT. (1 = Less mental energy than usual, 7 = More mental energy than usual)

Page Break

Thank you! Now please click --> to continue.

Page Break

**[Low reward condition]**


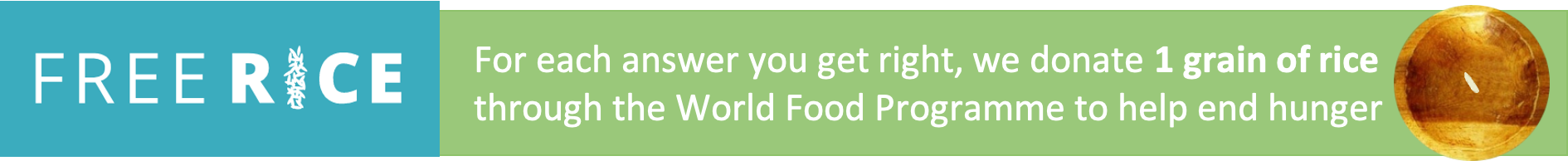
 **[High reward condition]**


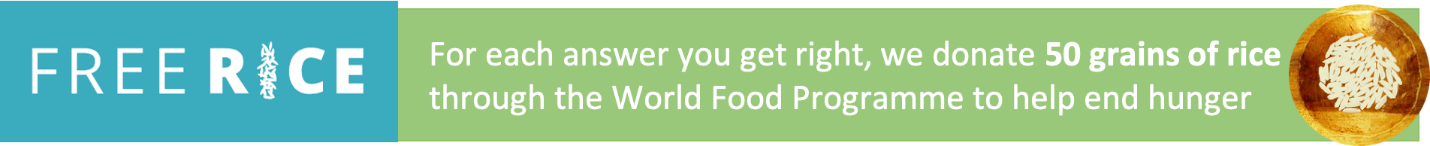


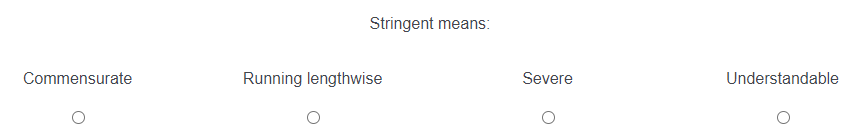


Page Break

**[Low reward condition]**


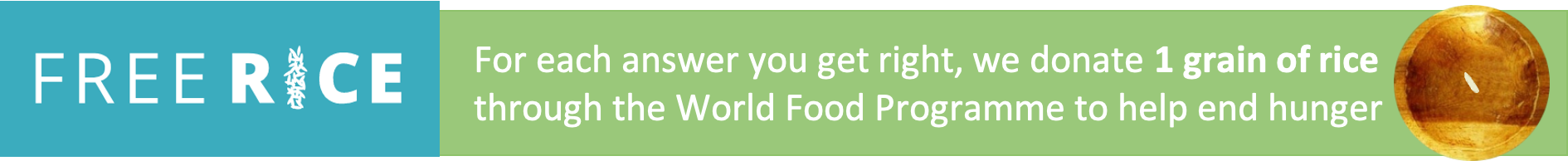
 **[High reward condition]**


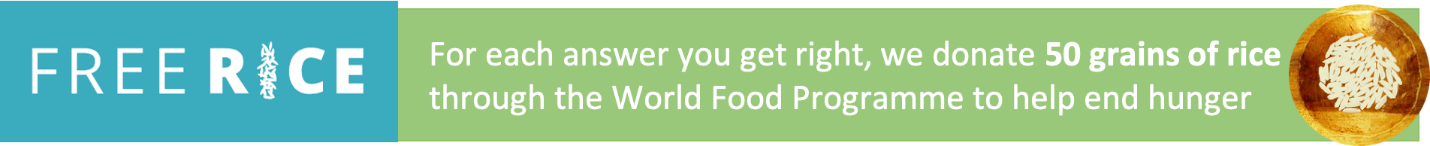


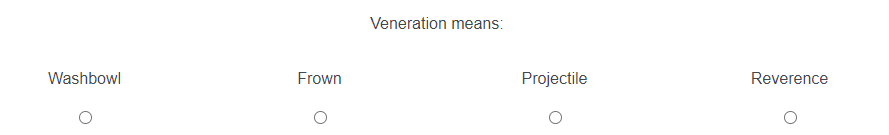


Page Break

**[Low reward condition]**


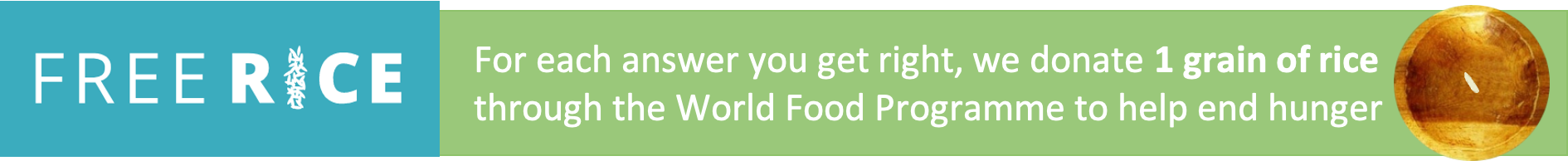
 **[High reward condition]**


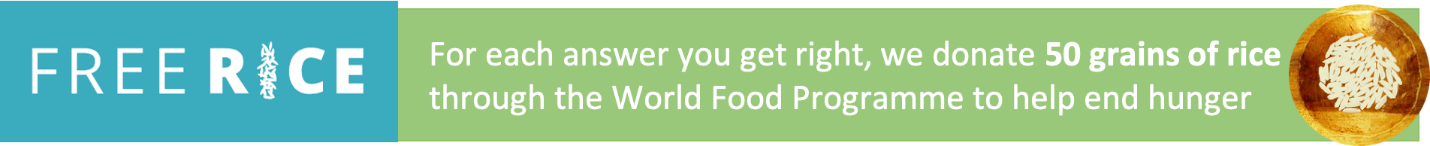


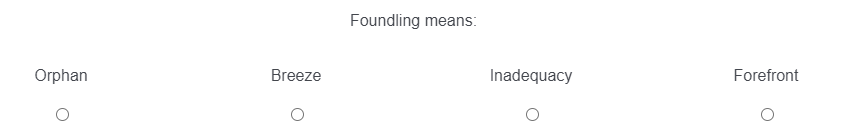


Page Break

**[Low reward condition]**


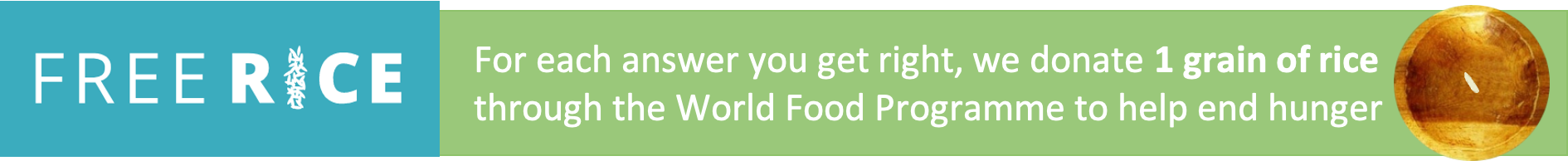
 **[High reward condition]**


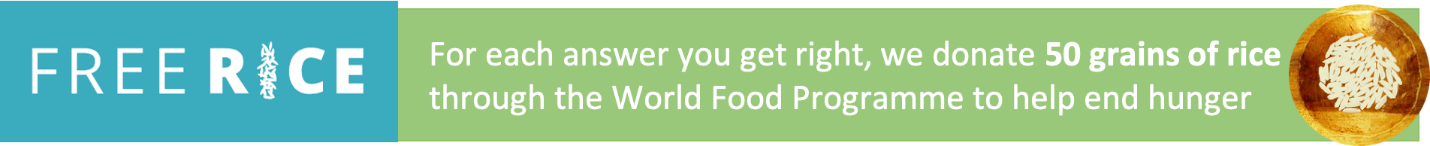


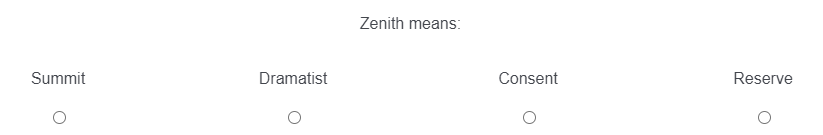


Page Break

**[Low reward condition]**


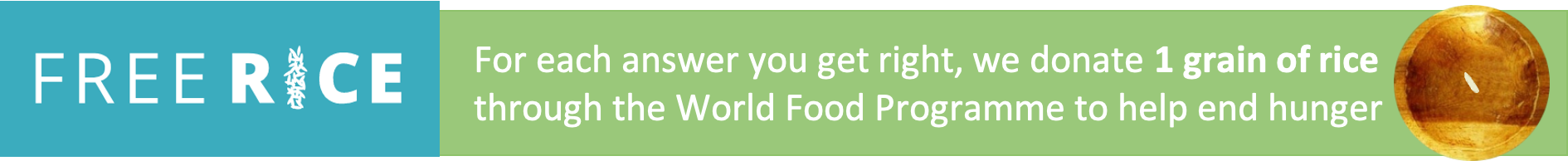
 **[High reward condition]**


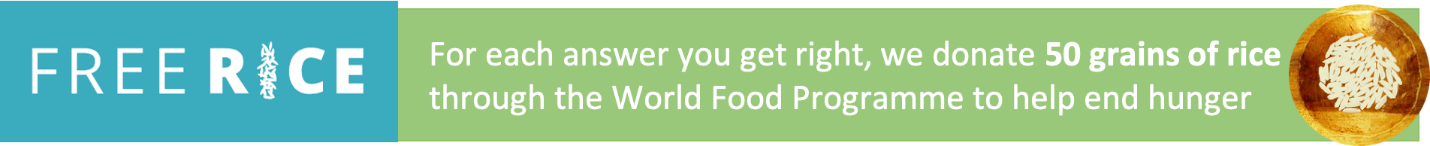


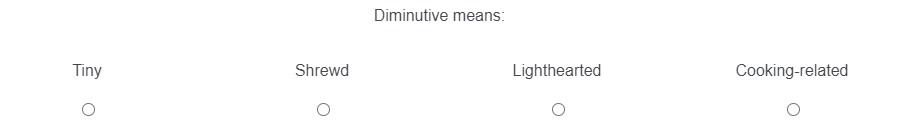


Page Break


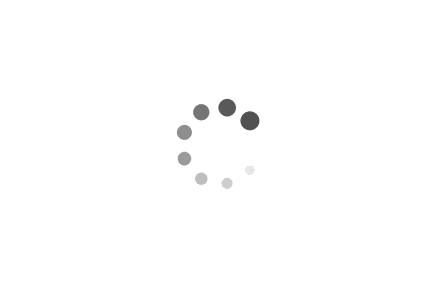


Loading the next question set

Page Break

[T2]

Before you continue, we would like to know how much mental energy you have at this moment. People's mental energy fluctuates on a moment-to-moment basis. On the following scale, please indicate how much mental energy you feel you have at this moment. (1 = Less mental energy than usual, 7 = More mental energy than usual)

Page Break

Thank you! Now please click --> to continue.

Page Break

**[Low reward condition]**


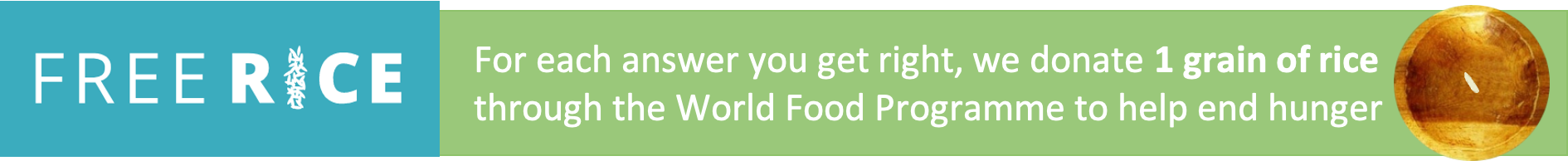
 **[High reward condition]**


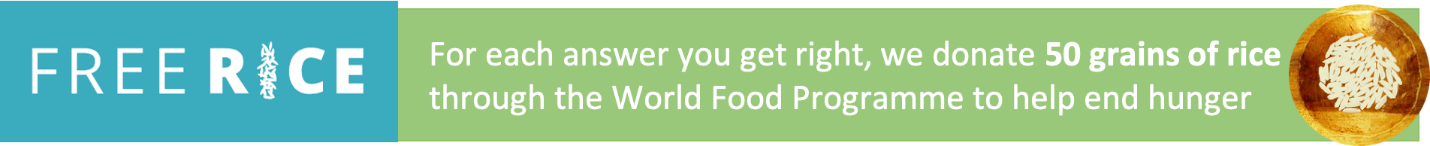


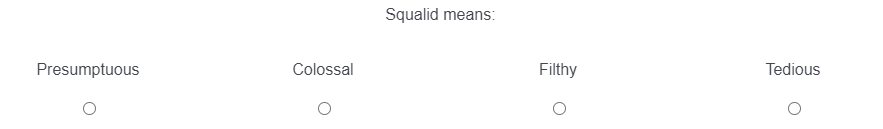


Page Break

**[Low reward condition]**


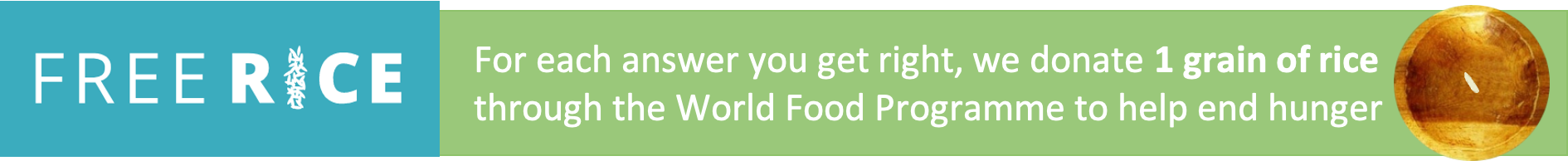
 **[High reward condition]**


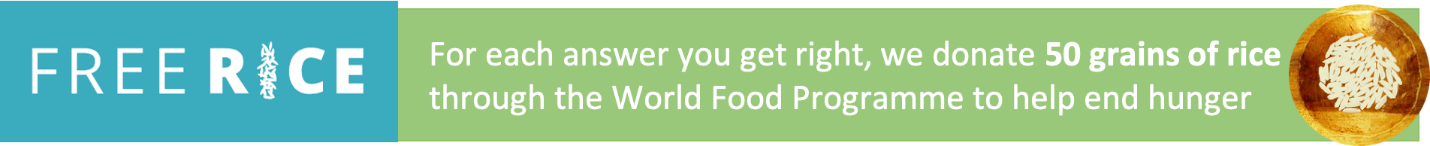


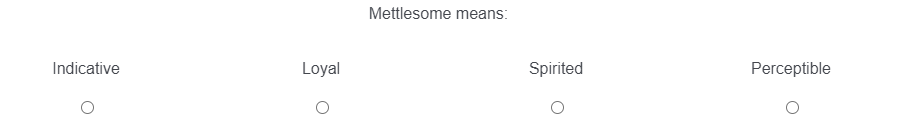


Page Break

**[Low reward condition]**


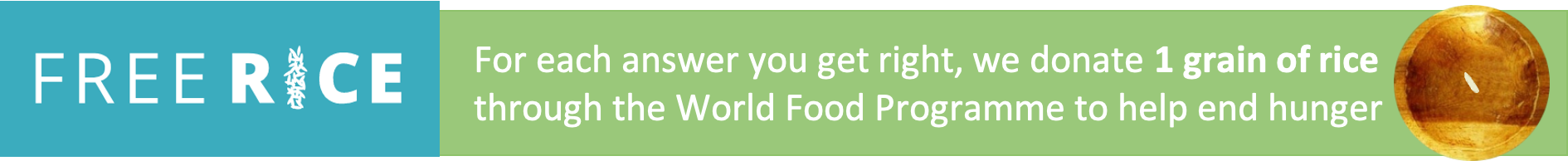
 **[High reward condition]**


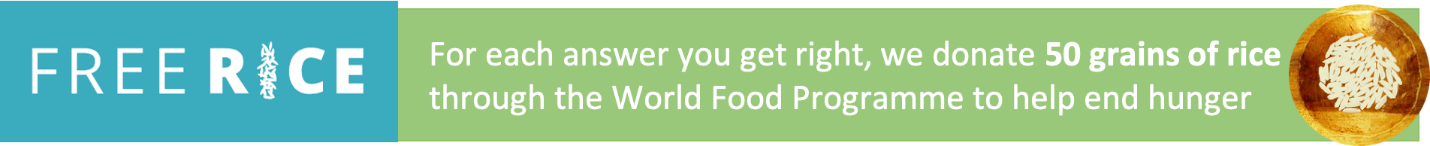


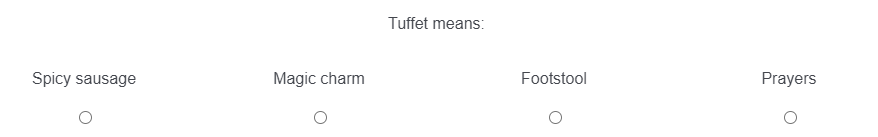


Page Break

**[Low reward condition]**


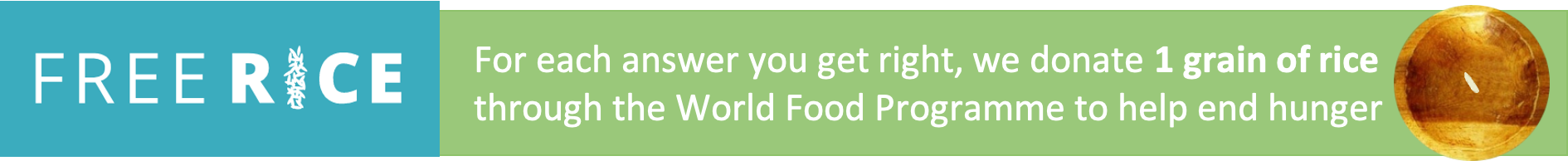
 **[High reward condition]**


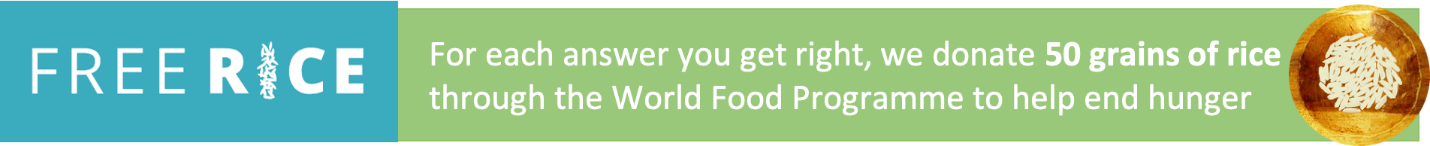


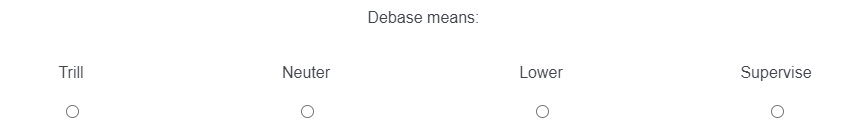


Page Break

**[Low reward condition]**


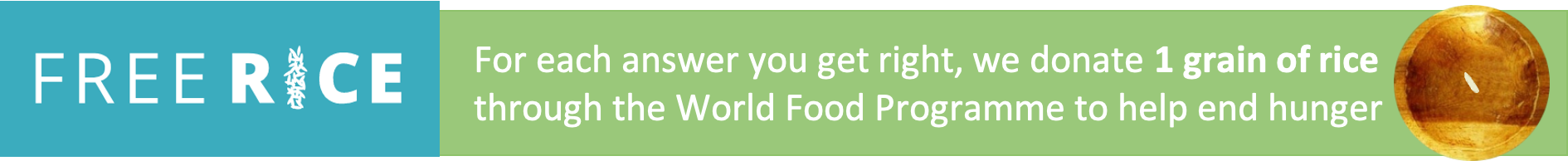
 **[High reward condition]**


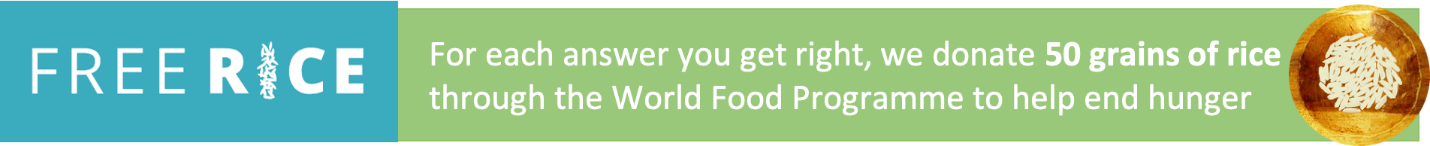


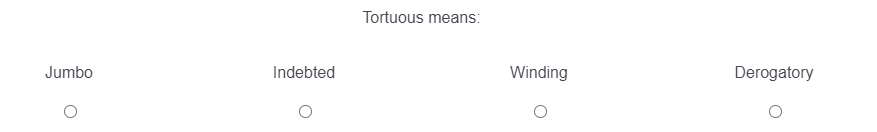


Page Break


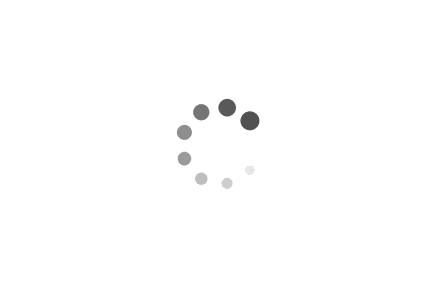


Loading the next question set

Page Break

[T3]

Before you continue, we would like to know how much mental energy you have at this moment. People's mental energy fluctuates on a moment-to-moment basis. On the following scale, please indicate how much mental energy you feel you have at this moment. (1 = Less mental energy than usual, 7 = More mental energy than usual)

Page Break

Thank you! Now please click --> to continue.

Page Break

**[Low reward condition]**


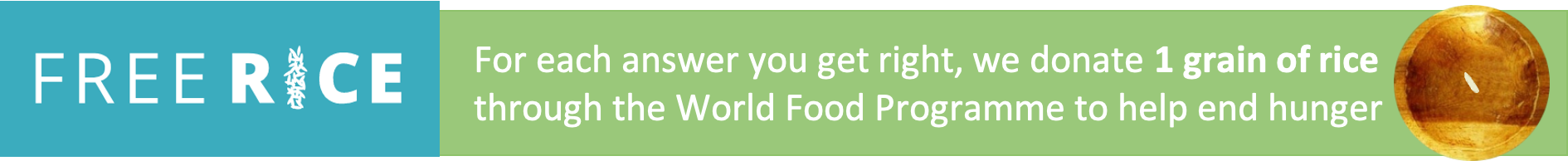
 **[High reward condition]**


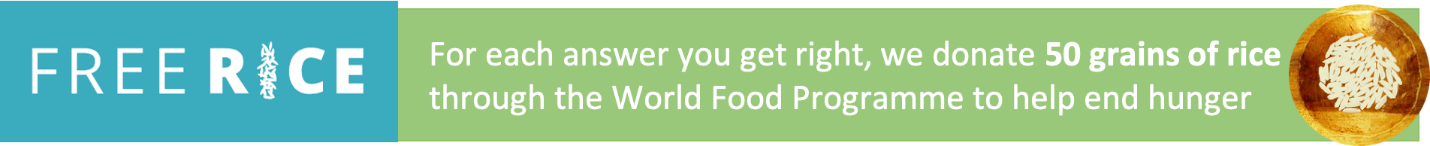


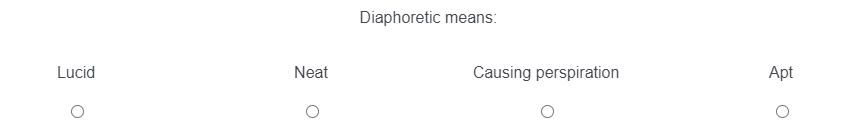


Page Break

**[Low reward condition]**


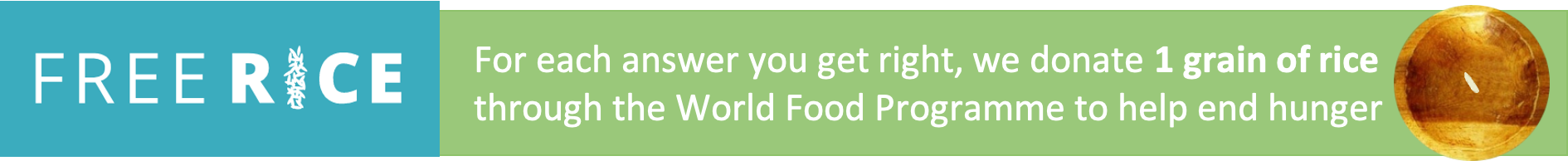
 **[High reward condition]**


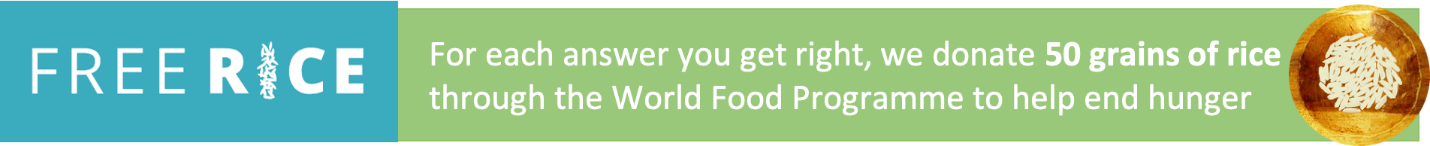


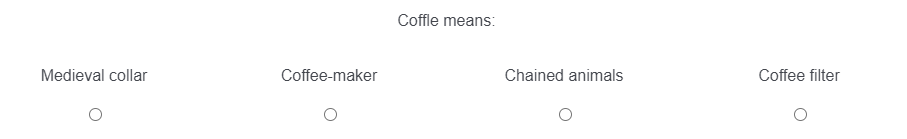


Page Break

**[Low reward condition]**


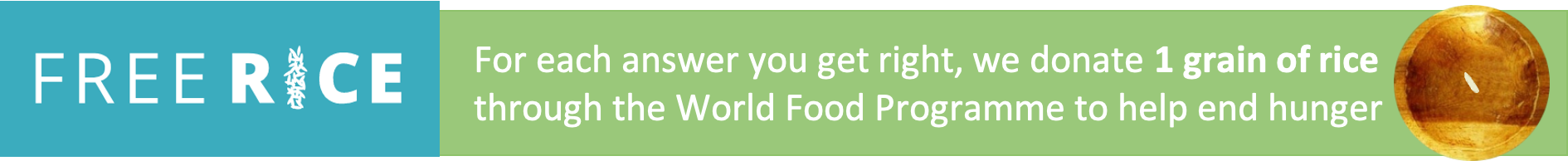
 **[High reward condition]**


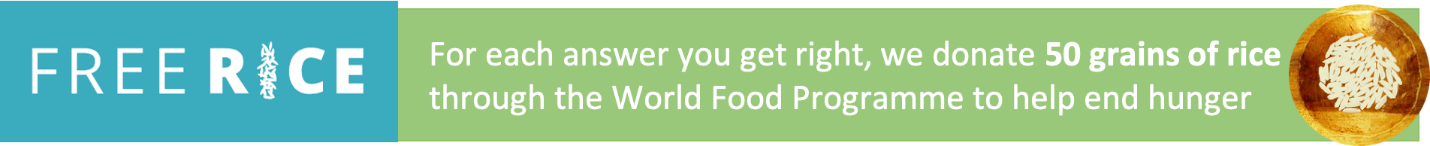


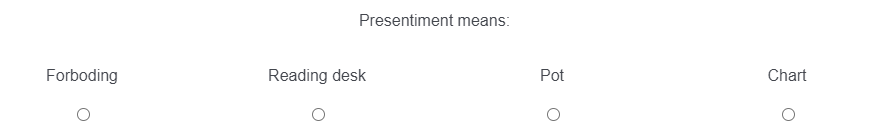


Page Break

**[Low reward condition]**


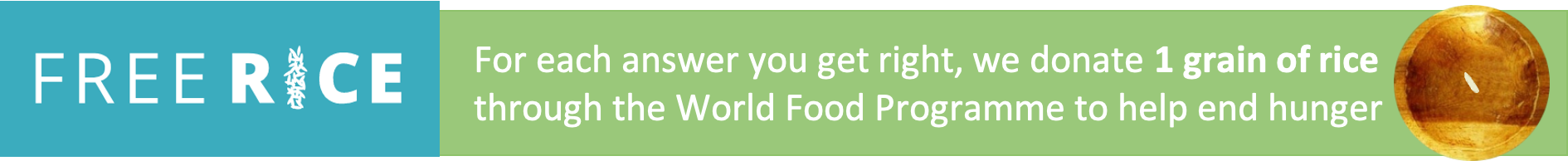
 **[High reward condition]**


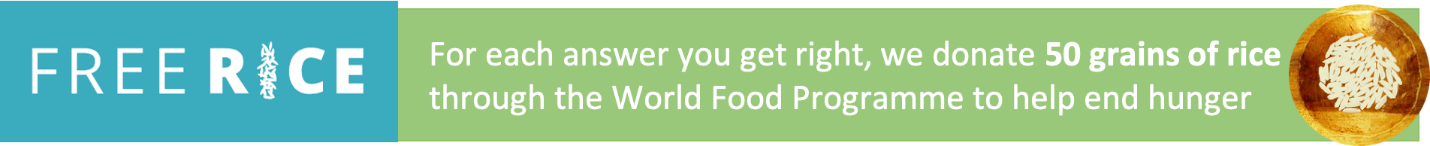


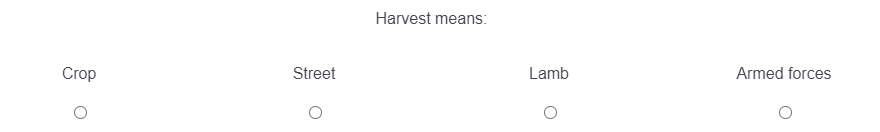


Page Break

**[Low reward condition]**


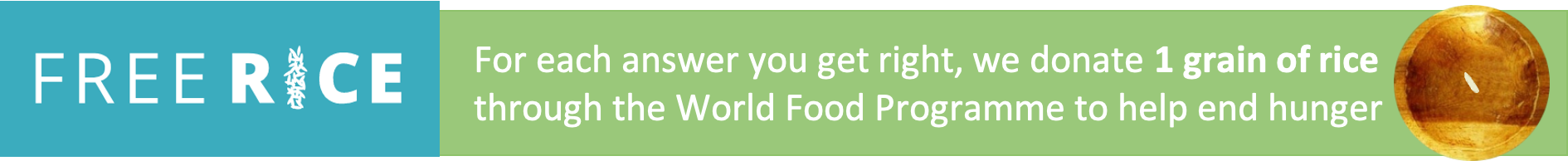
 **[High reward condition]**

Page Break

Loading the next question set

Page Break

[T4]

Before you continue, we would like to know how much mental energy you have at this moment. People's mental energy fluctuates on a moment-to-moment basis. On the following scale, please indicate how much mental energy you feel you have at this moment. (1 = Less mental energy than usual, 7 = More mental energy than usual)

Page Break

Thank you! Now please click --> to continue.

Page Break

**[Low reward condition]**

**[High reward condition]**

Page Break

**[Low reward condition]**

**[High reward condition]**

Page Break

**[Low reward condition]**

**[High reward condition]**

Page Break

**[Low reward condition]**

**[High reward condition]**

Page Break

**[Low reward condition]**

**[High reward condition]**

Page Break

Loading the next question set

Page Break

[T5]

Before you continue, we would like to know how much mental energy you have at this moment. People's mental energy fluctuates on a moment-to-moment basis. On the following scale, please indicate how much mental energy you feel you have at this moment. (1 = Less mental energy than usual, 7 = More mental energy than usual)

Page Break

Thank you! Now please click --> to continue.

Page Break

**[Low reward condition]**

**[High reward condition]**

Page Break

**[Low reward condition]**

**[High reward condition]**

Page Break

**[Low reward condition]**

**[High reward condition]**

Page Break

**[Low reward condition]**

**[High reward condition]**

Page Break

**[Low reward condition]**

**[High reward condition]**

Page Break

Congratulations! You have completed the task.

[T6]

On the following scale, please indicate how much energy you feel you have at this moment. (1 = Less mental energy than usual, 7 = More mental energy than usual)

Page Break

[Reward value manipulation check]

To what extent do you think the task was … (1 = not at all, 7 = very much)

- Valuable
- Important
- Rewarding
- Useful

Page Break

[Felt achievement measure]

To what extent do you think completing the task felt like an achievement? (1 = not at all, 7 = very much)

[Competence measure]

How effective did you feel at the task? (1 = not at all, 7 = very much)

# Study 4 Stimuli

2 (reward value: low vs. high) by 3 (actual effort: low vs. moderate vs. high)

**[High reward condition]**

Welcome!

In this HIT, we will show you some information tags of electronic products and would like you to transcribe the information into digital texts. Please transcribe exactly what you see on the tag.

You will receive an extra 5 cents for each tag you accurately transcribe.

Page Break

**[Low reward condition]**

Welcome!

In this HIT, we will show you some information tags of electronic products and would like you to transcribe the information into digital texts. Please transcribe exactly what you see on the tag.

You will receive an extra 1 cent for each tag you accurately transcribe.

Page Break

Loading the first tag...

Page Break

[T1]

Before you start, we would like to know how much mental energy you have at this moment.

**We need mental energy to get things done. Mental energy is how alert your brain feels at the moment – the degree to which you can remain focused on the very next task.

People’s mental energy fluctuates on a moment-to-moment basis. We will ask you to indicate how much mental energy you have at various time in this study.

On the following scale, please indicate how much mental energy you feel you have AT THIS MOMENT. (1 = Less mental energy than usual, 7 = More mental energy than usual)

Page Break

Thank you! Now please click >> to see the first tag.

Page Break

[Low actual effort condition]

[Moderate actual effort condition]

[High actual effort condition]

Please type the information in the text box below (press the "Enter" key after each line).

Page Break

Loading the next tag…

Page Break

[T2]

Before you continue, we would like to know how much mental energy you have at this moment. People's mental energy fluctuates on a moment-to-moment basis. On the following scale, please indicate how much mental energy you feel you have at this moment. (1 = Less mental energy than usual, 7 = More mental energy than usual)

Page Break

Thank you! Now please click --> to continue with the task.

Page Break

[Low actual effort condition]

[Moderate actual effort condition]

[High actual effort condition]

Please type the information in the text box below (press the "Enter" key after each line).

Page Break

Loading the next tag…

Page Break

[T3]

Before you continue, we would like to know how much mental energy you have at this moment. People's mental energy fluctuates on a moment-to-moment basis. On the following scale, please indicate how much mental energy you feel you have at this moment. (1 = Less mental energy than usual, 7 = More mental energy than usual)

Page Break

Thank you! Now please click --> to continue with the task.

Page Break

[Low actual effort condition]

[Moderate actual effort condition]

[High actual effort condition]

Please type the information in the text box below (press the "Enter" key after each line).

Page Break

Loading the next tag…

Page Break

[T4]

Before you continue, we would like to know how much mental energy you have at this moment. People's mental energy fluctuates on a moment-to-moment basis. On the following scale, please indicate how much mental energy you feel you have at this moment. (1 = Less mental energy than usual, 7 = More mental energy than usual)

Page Break

Thank you! Now please click --> to continue with the task.

Page Break

[Low actual effort condition]

[Moderate actual effort condition]

[High actual effort condition]

Please type the information in the text box below (press the "Enter" key after each line).

Page Break

Loading the next tag…

Page Break

[T5]

Before you continue, we would like to know how much mental energy you have at this moment. People's mental energy fluctuates on a moment-to-moment basis. On the following scale, please indicate how much mental energy you feel you have at this moment. (1 = Less mental energy than usual, 7 = More mental energy than usual)

Page Break

Thank you! Now please click --> to continue with the task.

Page Break

[Low actual effort condition]

[Moderate actual effort condition]

[High actual effort condition]

Please type the information in the text box below (press the "Enter" key after each line).

Page Break

Congratulations! You have completed the task.

[T6]

On the following scale, please indicate how much energy you feel you have at this moment. (1 = Less energy than usual, 7 = More energy than usual)

Page Break

[Affect measure]

How happy do you feel right now? (1 = not at all, 7 = very much)

Page Break

[Felt achievement measure]

To what extent do you think completing the task felt like an achievement? (1 = not at all, 7 = very much)

[Competence measure]

How effective did you feel at the task? (1 = not at all, 7 = very much)

Page Break

[Reward value manipulation check]

How rewarding do you think the extra payment for accurately transcribing the information tags is? (1 = not at all, 7 = very much)

# Study 4 Results: Means of T1 through T6
